# Forest under stress: responses and recovery of water relations across scales during the compound drought in summer 2022

**DOI:** 10.1111/nph.71516

**Published:** 2026-09-04

**Authors:** Ankit Shekhar, Anna K. Gilgen, Annika Ackermann, Liliana Scapucci, Sergio Aranda‐Barranco, Anastasiia Bolshakova, Lukas Hörtnagl, Yi Wang, Franziska Richter, Fabio Turco, Ruikun Gou, Luana Krebs, Iris Feigenwinter, Roland A. Werner, Roman Zweifel, Sophia Etzold, Marius Floriancic, Stefan Osterwalder, Mana Gharun, Nina Buchmann

**Affiliations:** ^1^ Agricultural and Food Engineering Department IIT Kharagpur 721302 Kharagpur India; ^2^ Institute of Agricultural Sciences ETH Zurich 8092 Zurich Switzerland; ^3^ Andalusian Institute for Earth System Research University of Granada 18006 Granada Spain; ^4^ Department of Civil Engineering, Lassonde School of Engineering York University M3J 1P3 Toronto Canada; ^5^ ICOS ERIC Carbon Portal, Department of Earth and Environmental Sciences Lund University 22362 Lund Sweden; ^6^ Swiss Federal Institute for Forest Snow and Landscape Research (WSL) 8903 Birmensdorf Switzerland; ^7^ College of Forestry Northwest A&F University Yangling 712100 China; ^8^ Department of Earth System Science Stanford University Stanford 94305 CA USA; ^9^ Department of Civil, Environmental and Geomatic Engineering ETH Zurich 8903 Zurich Switzerland; ^10^ Department of Geography University of Zurich 8057 Zurich Switzerland; ^11^ Faculty of Geosciences University of Münster 48149 Münster Germany

**Keywords:** beech, eddy covariance, evapotranspiration, fir, leaf water potential, maple, spruce, stable water isotopes

## Abstract

- The summer of 2022 was characterized by compound soil and atmospheric drought (CSAD; low soil water content, SWC, and high vapor pressure deficit, VPD) in Central Europe, threatening forest carbon sequestration and water balance, yet linked ecosystem‐ and tree‐level hydraulic responses remain poorly resolved.
- We assessed the response and recovery of water relations of a beech‐dominated montane mixed deciduous forest in Switzerland (CH‐Lae) to the 2022 CSAD through a series of ecosystem‐scale (evapotranspiration, ET; canopy conductance, Gs) as well as tree‐scale measurements, including leaf‐level (leaf water potential, LWP; stomatal conductance, gs), stem‐level (nighttime stem rehydration, NSR) and root‐level (root water uptake depth, RWU depth) variables.
- During peak CSAD, ET and Gs declined by up to 50%, driven primarily by low SWC. Midday and predawn LWP declined significantly. Beech and maple accessed deeper water (> 70 cm), maintaining NSR and gs during CSAD and fully recovering thereafter; spruce and fir relied on shallow water (< 30 cm), showing stronger NSR reductions but still full recovery post‐CSAD.
- Our findings demonstrate that species‐specific hydraulic traits drive forest‐scale water dynamics under CSAD conditions. Integrating these insights into forest adaptation strategies will be critical for sustaining forests under intensifying compound droughts in the future.

## Graphical abstract

A schematic showing the links of tree‐scale (root‐, stem‐, and leaf‐level) and ecosystem‐scale water relations during the CSAD at CH‐Lae.

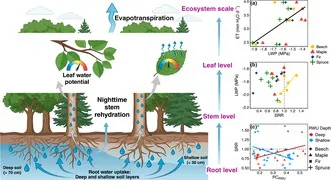

## Introduction

In recent years, Europe has experienced a significant increase in frequency and intensity of compound soil and atmospheric drought (CSAD) events (Shekhar *et al*., 2024a), which are characterized by simultaneous occurrence of low soil water contents (SWC) and high vapor pressure deficits (VPD; Scapucci *et al*., 2024). The summers of 2003, 2015, 2018, and more recently 2022 were characterized by CSAD conditions across Europe (Ionita *et al*., 2017, 2021; Dirmeyer *et al*., 2021; Tripathy & Mishra, 2023; Gharun *et al*., 2024). Occurrence of such CSAD events is projected to increase in the future (Zhou *et al*., 2019; Shekhar *et al*., 2024a), thereby posing a significant threat to temperate forest ecosystems, which are vital carbon sinks and play an important role in regulating global and regional water cycles (Harris *et al*., 2021; Anderegg *et al*., 2022). While the impact of CSAD on forest carbon relations (e.g. carbon assimilation and respiration) has received considerable attention (Bastos *et al*., 2020; Gharun *et al*., 2020; Xu *et al*., 2020; Dannenberg *et al*., 2022; Scapucci *et al*., 2024), the consequences of CSAD on forest water relations have been less extensively studied. Water relations, including ecosystem‐scale evapotranspiration (ET) and surface canopy conductance (Gs), as well as tree‐ and leaf‐level processes, such as leaf stomatal conductance (gs), nighttime stem rehydration (NSR), and root water uptake (RWU) among others, are critical for maintaining ecosystem functioning and tree vitality, particularly during and after CSAD events. Understanding how forested ecosystems deal with water limitations and how they recover their water balance after CSAD events is key to predicting their resilience to future climate extremes (Hesse *et al*., 2023).

Ecosystem‐scale ET and Gs, a canopy analog to leaf‐level gs, are crucial indicators of water dynamics of forest ecosystems. Past studies have shown that both ET and Gs are sensitive to soil and atmospheric drought stress (Granier *et al*., 2007; Aranda *et al*., 2012; Hesse *et al*., 2023). Soil drought limits water availability for roots, leading to reduced transpiration, which is the major component of ET in forests (Paul‐Limoges *et al*., 2020; Shekhar *et al*., 2024b). Atmospheric drought, characterized by high water vapor deficit (VPD), initially enhances both evaporation and transpiration due to increased atmospheric water demand, but often leads to reduced gs when combined with soil drought, eventually leading to reduced ET (Aranda *et al*., 2012; He *et al*., 2022). Postdrought recovery, that is percent change relative to predrought conditions, is often gradual and variable, depending on plant species‐specific ecophysiological and morphological traits and drought severity. Rehydration of soil and xylem is essential for recovery, but stomatal reopening remains conservative to prevent hydraulic failure, leading to a lag in ET recovery (Anderegg *et al*., 2014; Taborski *et al*., 2022). Long‐term recovery of Gs might be limited by structural changes, such as leaf area reductions or xylem damage, which hinder water flux recovery (Diao *et al*., 2021). These ecosystem‐scale dynamics reflect underlying tree‐level responses. However, which individual hydraulic traits and stomatal behavior determine the resistance (response during extreme conditions) and recovery (response postextreme conditions) of water relations of forests is less clear.

Tree‐scale water relations during drought involve interacting responses at leaf‐, stem‐ and root‐level. These include changes in LWP, gs, NSR, and RWU depth. Drought‐induced declines in predawn (LWP_pd_) and midday (LWP_md_) leaf water potentials (LWP) reflect the interplay between limited soil moisture availability and high atmospheric demand, with LWP_md_ reaching more negative values due to pronounced midday evaporative stress (Ruehr *et al*., 2019; Fang *et al*., 2021). Stomatal closure during drought reduces gs to mitigate water loss but also reduces CO_2_ uptake, thereby creating a trade‐off between hydraulic safety and photosynthesis (Bonan *et al*., 2014; Wehr & Saleska, 2021). Species‐specific stomatal sensitivity to VPD further regulates gs recovery, with drought‐tolerant species showing earlier stomatal reopening to resume photosynthesis (Berdanier & Clark, 2018; Wehr & Saleska, 2021; Martinetti *et al*., 2025). Moreover, during the day, the water loss via transpiration is larger than the replenishment of the water via roots, resulting in stem shrinkage (Zweifel *et al*., 2009). At night, when stomata are mostly closed and transpiration is strongly reduced, trees rely on NSR to replenish water lost during the day and restore hydraulic functioning (Peters *et al*., 2023). This strong relationship between daytime gs and NSR is critical for restoring xylem hydraulic capacity during severe soil droughts. Ultimately, RWU depth plays a key role in shaping tree‐ and leaf‐level water relations during CSAD. Deep‐rooted species, such as beech (*Fagus sylvatica* L.), access subsurface water, enabling faster recovery of LWP_pd_ and sustained NSR, which facilitates gs (Berdanier & Clark, 2018; Brinkmann *et al*., 2019; Hesse *et al*., 2023). By contrast, trees with shallow roots are more dependent on surface soil moisture, which decreases quickly during dry periods. This limited water availability at shallow soil depths compromises NSR, leading to progressively lower gs and LWP as the drought persists.

Despite our conceptual understanding of these individual underlying mechanisms, the complexity of interacting responses makes predictions of forest water dynamics under CSAD conditions challenging, especially in a mixed forest setting. Moreover, these interactions between stomatal behavior, LWP, NSR, and shifts in RWU depth reflect a tree's capability to balance water loss and uptake across varying environmental conditions (Brinkmann *et al*., 2019; Hesse *et al*., 2023; Peters *et al*., 2023). Especially in species‐rich forests, such species‐specific hydraulic traits ensure survival and maintenance of productivity of tree species in mixed forests under extreme climate.

Most of the studies have focused on tree‐scale responses (e.g. gs, NSR, and RWU depth) or ecosystem‐scale responses (e.g. ET) in isolation, with few studies highlighting how species‐specific hydraulic strategies aggregate to drive total forest water relations during compound drought conditions. The severe 2022 CSAD conditions across central Europe threatened forest ecosystem functioning (Gharun *et al*., 2024; Scapucci *et al*., 2024), thereby providing an opportunity to assess the response and the recovery of ecosystem‐scale and tree‐scale water relations of a beech‐dominated mixed deciduous forest in Switzerland (CH‐Lae). The objective of this study was to assess the tree‐scale physiological responses and ecosystem‐scale fluxes by evaluating how species‐specific traits across the hydraulic spectrum determine the stand‐level resistance (i.e. response during the CSAD) and recovery (i.e. response post‐CSAD) of the forest to a CSAD event at a deciduous mixed forest (CH‐Lae). We tested three primary hypotheses:

1. Hypothesis 1: ecosystem‐scale regulation of water fluxes during compound soil and atmospheric drought (CSAD) is directly dependent on the leaf‐level physiological responses.
2. Hypothesis 2: the link between maintenance of leaf‐level gas exchange (gs) and leaf water potential (LWP) to nighttime stem rehydration (NSR) is dependent on species‐specific hydraulic strategies.
3. Hypothesis 3: the capacity for nighttime stem rehydration (NSR) is determined by root‐level responses, with the plasticity of tree species to shift root water uptake (RWU) to deeper soil layers (> 70 cm) enabling stem water rehydration despite rapid soil drying.

## Materials and Methods

### Study site

The study was carried out in a montane mixed deciduous forest (CH‐Lae as part of FLUXNET and Swiss FluxNet, https://www.swissfluxnet.ethz.ch/index.php/sites/site‐info‐ch‐lae/) situated at 682 m above sea level within the Lägeren forest, part of the Jura Mountain range (limestone bedrock) in Switzerland. CH‐Lae is the only mixed forest in Switzerland where eddy covariance (EC) observations are available. This managed forest has a high species diversity and thus a complex canopy structure, predominantly consisting of European beech (*Fagus sylvatica* L., beech hereafter), along with European ash (*Fraxinus excelsior* L., ash hereafter), sycamore maple (*Acer pseudoplatanus* L., maple hereafter), European silver fir (*Abies alba* Mill., fir hereafter), and Norway spruce (*Picea abies* (L.) Karst., spruce hereafter; Ruehr & Buchmann, 2010). The forest understory is dominated by wild garlic (*Allium ursinum* L.), growing for a short period from March through June (Ruehr & Buchmann, 2010; Scapucci *et al*., 2025). The soils at CH‐Lae are predominantly Rendzic Leptosols and Haplic Cambisols, underlain by bedrock consisting of limestone, marls, sandstone, and transitional zones between these formations (Ruehr *et al*., 2010) The site has an average annual temperature of 8.8°C and receives *c*. 842 mm of annual precipitation, based on data for the period 2004–2022.

### Ecosystem‐scale measurements

We used continuous measurements of water vapor (H_2_O) fluxes at ecosystem‐scale based on the eddy covariance (EC) technique (Aubinet *et al*., 2012) between 2004 and 2022 to estimate ecosystem evapotranspiration (ET). The EC system (eddy tower coordinates: 47°28′42.0″N and 8°21′51.8″E) was installed at a height of 47 m (mean canopy height of 30 m). We measured wind speed and wind direction using a 3D sonic anemometer (Model HS‐50; Gill Instruments Ltd, Lymington, UK), and H_2_O concentration using an infrared gas analyzer (model LI‐7500 (2004–2015) and model LI‐7200 (2016–2022); Li‐Cor Inc., Lincoln, NE, USA), at a frequency of 20 Hz. Half‐hourly fluxes of H_2_O (*F*_W_, mmol H_2_O m^−2^ s^−1^) were calculated from the 20 Hz measurements by calculating the covariance of vertical wind speed and concentration of H_2_O, using the Eddypro software (v.7.0.9; Li‐Cor Inc.) following established community guidelines (Aubinet *et al*., 2012; Sabbatini *et al*., 2018). The time lag of H_2_O concentrations (molar density using LI‐7500, and mixing ratio using LI‐7200) was calculated by the covariance maximization method (Fan *et al*., 1990). *F*_W_ were filtered for turbulent conditions based on the steady‐state statistic and integral turbulence characteristics test (Foken *et al*., 2004; Mauder & Foken, 2006). An additional quality control flag (QCF) for each half‐hourly H_2_O flux was calculated based on Sabbatini *et al*. (2018) to remove any fluxes with QCF = 2 (unreliable flux value). The latent heat flux (LE, W m^−2^), which is an energy flux equivalent of F_W_, underwent absolute threshold filtering of −200 to 1000 Wm^−2^. For more information on the quality check and filtering of *F*_W_, see Shekhar *et al*. (2024b). Thereafter, the missing and filtered LE (based on the above‐mentioned quality control) was gap‐filled by the marginal distribution sampling (MDS) approach using the reddyproc R‐package (Reichstein *et al*., 2005; Wutzler *et al*., 2018). Readers should note that no friction velocity (USTAR) filtering was done for *F*_W_, as suggested by Pastorello *et al*. (2020). The gap‐filled LE was converted to ET (mm h^−1^) for further analysis based on temperature‐dependent latent heat of vaporization. The final EC dataset is available from Hörtnagl *et al*. (2023). Along with ET, we also measured half‐hourly air temperature (Tair) and relative humidity (RH; Rotronic MP101 A, Rotronic AG, Bassersdorf, Switzerland), incoming short‐wave radiation (SWin; BF2_BF2116, Delta‐T Devices Ltd, Cambridge, UK) and precipitation (LAMBRECHT Meteo GmbH, Göttingen, Germany) at the top of the EC tower from 2004 to 2022. We estimated half‐hourly vapor pressure deficit (VPD) from half‐hourly measurements of Tair and RH (see Shekhar *et al*., 2024b). We also measured continuous soil water content (SWC) at different depths (Decagon ECH2O EC‐5; Pullman, WA, USA, from 2004 to 2019, and TEROS 12; METER Group Pullman, WA, USA, from 2020 onwards at 5, 10, 20, 30, and 50 cm). Due to data gaps for different soil depths, we only used SWC measured at 20 cm depth (as it had shortest gaps), which showed a similar soil drought pattern as that of deeper soil level (30 and 50 cm). We installed a new soil moisture profile (TEROS 12; METER Group Pullman, WA, USA) in 2020, which showed different absolute values compared with the previously measured (2004–2019) SWC. Therefore, we normalized SWC of 2004–2019 and 2020–2022 (based on mean and SD), and corrected the latter based on the mean obtained from the 2004–2019 data (Supporting Information Fig. S1).

To study the intra‐annual variability of ET, VPD and SWC of 2022, we compared the 5‐d moving daily means (assigned to the center of the 5‐d period) with those of the reference period (2004–2021; also 5‐d moving daily means). We identified the compound soil and atmospheric drought (CSAD) period in 2022 as period with more than 10 consecutive days when SWC was significantly lower (*P* < 0.05) and VPD was significantly higher (*P* < 0.05) than those of the reference period (Scapucci *et al*., 2024) as well as daily precipitation of < 4 mm (mean summer daily precipitation at CH‐Lae over 2004–2022). Thereafter, we divided the summer of 2022 into three periods: pre‐CSAD, CSAD, and post‐CSAD.

To investigate the drivers (SWin, VPD, SWC, and wind speed) of ecosystem‐scale ET, we employed an explainable machine learning approach. Specifically, we used a tree‐based ensemble‐learning eXtreme Gradient Boosting (XGB) model (Chen & Guestrin, 2016) combined with a TreeExplainer‐based SHapley Additive exPlanations (SHAP) framework (Lundberg & Lee, 2017; Lundberg *et al*., 2020). The XGB model was used to predict daily mean ET, with its hyperparameters (number of trees and initial learning rate) optimized using the GridSearchCV method (for details, see Wang *et al*., 2022). The SHAP framework, integrated with the XGB model, applies game theory (Shapley, 1953) to estimate the marginal contribution (SHAP value) of each predictor variable (e.g. daily means of SWin, VPD, and SWC) to simulated ET. A positive SHAP value indicates that the specific value of a predictor variable for a given day results in an increase in the response variable (here ET) from its overall mean (mean ET from 2005 to 2022), and vice versa. The models were built separately for each year, enabling us to analyze the daily temporal variation in the marginal contributions of predictors to better account for the long‐term variability in ET. The mean *R* ^2^ and root mean square error (RMSE) of the XGB model across all years were 0.82 and 0.32 mm d^−1^, respectively. Additionally, we calculated the mean SHAP values for 2022 and compared them with the reference period (2004–2021) to identify deviations from the long‐term mean to quantify the drought effect. This approach provided insights into temporal patterns and the relative influence of each predictor on ET in typical non‐CSAD years and in the 2022 CSAD. For the above‐mentioned machine learning analysis, we used the R package xgboost (Chen & Guestrin, 2016) and shapforxgboost (Chen & Guestrin, 2016).

To understand the ecosystem‐scale stomatal response of the canopy to VPD during the CSAD event, we calculated surface canopy conductance (Gs; m s^−1^) by inverting the Penman–Monteith equation (Monteith, 1965). Gs has physiological significance only when vegetation is active and when evaporation from surfaces is limited, that is during such times Gs ≈ canopy conductance (Zhou *et al*., 2014). Therefore, we removed data of the 2 d following precipitation events if daily precipitation exceeded 0.5 mm to minimize the effect of evaporation on Gs after rainfall. We calculated half‐hourly Gs using the bigleaf R‐package (Knauer *et al*., 2018) and aggregated it to daily averages to calculate the Gs sensitivity to daily mean VPD (Oren *et al*., 1999; Gharun *et al*., 2015) as shown in Eqn 1.

(Eqn 1) $$\mathrm{Gs}=-m*\log_{e}\left(\mathrm{VPD}\right)+b$$

The Gs sensitivity to VPD is indicated by the parameter *m* (units: m s^−1^ log_e_(kPa)^−1^) in Eqn 1. The parameter ‘*m*’ is the slope of the linear regression between Gs and log_e_(VPD). We further compared the Gs sensitivity to VPD (i.e. ‘*m*’) of 2022 with that of the reference period (2004–2021). A higher *m* value indicates a higher dependence of Gs on VPD, and vice versa.

### Tree‐ and leaf‐level measurements

#### Leaf water potential and stomatal conductance

Along with ecosystem‐scale measurements of evapotranspiration (ET) and the calculation of Gs, we conducted tree‐ and leaf‐level measurement campaigns at the Lägeren forest site on 7 d during July to September 2022 (14, 16, 19 and 22 July; 4 and 11 August; 23 September). For leaf‐level measurements, we selected 14 tree individuals, including six beech, two maple, three spruce, and three fir trees. The selected trees were fully developed and were growing within the footprint area of the eddy covariance (EC) flux tower. Trees were between 100 and 150 yr old, dbh varied between 30 and 80 cm, and tree height between 15 and 25 m. During these seven measurement campaigns, we measured predawn leaf water potential (LWP_pd_) between 04:00 h and 05:00 h, and midday leaf water potential (LWP_md_) between 13:00 h and 15:00 h of all individuals of the four tree species, as well as midday gs of broadleaf trees (i.e. beech and maple). We cut tree branches from 8 to 12 m height in the canopy using a telescopic pruner and measured LWP and gs within 2 min on at least three leaves per branch. During midday, we sampled sun‐exposed branches from a similar location in the canopy as for the predawn measurements. We measured LWP (MPa) using a Scholander‐type (Scholander *et al*., 1965) pressure chamber (Model 600; PMS, Corvallis, OR, USA) and gs (mol m^−2^ s^−1^) using a LI‐600 Porometer (Li‐Cor Inc., Lincoln, NE, USA). To understand leaf‐level stomatal sensitivity to VPD, we fitted gs to VPD (mean of midday VPD, measured at the EC tower) using Eqn 1 (gs instead of Gs). In addition to LWP measurements during the 2022 field campaigns, routine LWP measurements were performed at the site in 2020 and 2021 on the same four tree species. We used the LWP measurements of 2020 and 2021, which were nondrought years at the site (non‐CSAD years; Scapucci *et al*., 2024), to compare with the 2022 CSAD measurements. To understand the effect of VPD and SWC on LWP, we used a linear regression model with interaction effect (as SWC and VPD are correlated). The linear regression model is shown in Eqn 2.

(Eqn 2) $$\mathrm{LWP}=a0+a1.\mathrm{SWC}+a2.\mathrm{VPD}+a3.\left(\mathrm{SWC}\times \mathrm{VPD}\right)$$

The model estimates *a*1 and *a*2 show the main effect of SWC and VPD on LWP, whereas *a*3 shows the interaction effect of SWC and VPD on LWP.

#### Nighttime stem rehydration estimates

In addition to leaf‐level measurements, we used continuous measurements of stem radius using high‐precision (resolution *c*. 1 μm) point dendrometers (ZN11‐T‐IP and ZN11‐T‐WP; Natkon, Oetwil am See, Switzerland). For 2022, stem radius data from 11 dendrometers were used (three each on beech, spruce, and fir; two on maple). The dendrometers (one per tree) were mounted at breast height on the tree stem (see Shekhar *et al*., 2024b for more information). Stem radius was measured every 30 s and subsequently averaged to 10‐min averages using a data logger (CR1000; Campbell Scientific Inc., Logan, UT, USA). The 10‐min raw dendrometer data were processed using the treenetproc R package (Haeni *et al*., 2020; Knüsel *et al*., 2021). The dendrometer data capturing stem radius variations reflect two distinct physiological processes: (1) irreversible stem growth (GRO, μm) and (2) reversible water‐driven stem swelling and shrinkage, resulting from rehydration and dehydration of wood and bark tissue (Zweifel *et al*., 2016). During periods without stem growth, changes in stem radius result from daytime stem dehydration (DSD, μm) due to water losses through transpiration and from nighttime stem rehydration (NSR) (μm; Fig. S2) due to root water uptake. We calculated the stem rehydration ratio (SRR) as the ratio of NSR and DSD (Fig. S2). Thus, SRR ≥ 1 implies full rehydration of the stems, whereas SRR < 1 implies increased tree water deficit. To understand the effects of the 2022 CSAD event on stem rehydration, we studied pre‐CSAD, CSAD, and post‐CSAD variations of SRRs for all individual trees of the four species.

#### Stable water isotope measurements of soil water and xylem water

We collected bulk soil (for soil water extraction), tree branch (for xylem water extraction) samples and precipitation water at the site to estimate root water uptake (RWU) depth based on the stable water isotopic signatures (δ^18^O and δ^2^H) of soil and xylem water. For soil water, we randomly sampled seven locations around the area where we measured the tree‐level LWP and gs. We collected soil samples down to 70 cm depth using a Pürkhauer soil corer with a diameter of 28 mm (Goecke, Schwelm, Germany). The soil cores were divided into seven different soil depths (0–10, 10–20, 20–30, 30–40, 40–50, 50–60, and 60–70 cm). Soil samples from each layer were stored in 12‐ml airtight glass exetainers (Labco Ltd, Lampeter, UK) and stored on ice in the field until placed in a freezer at −23°C before soil water extraction. The tree branches (1–2 cm diameter) were sampled from the same tree branches used for LWP and gs measurements, that is from 14 trees of four species. The bark and phloem were manually removed from twig samples and the remaining xylem was stored similar to the soil samples. We extracted water from soil and xylem samples using a cryogenic water extraction line (Ehleringer *et al*., 2000). Samples in the 12‐ml glass exetainers were immersed in an 80°C water bath. The evaporated water was collected in U‐tubes submerged in liquid nitrogen (−196°C) for condensation. Extraction was conducted under a vacuum of *c*. 0.03 hPa for 2 h. After extraction, the U‐tubes were sealed and allowed to thaw; the condensed water was collected and stored in 1.5‐ml airtight glass vials (Macherey‐Nagel, Düren, Germany) until isotope analysis.

Measurements of stable oxygen and hydrogen isotope ratios (δ^18^O and δ^2^H, respectively) were performed using a high‐temperature conversion/elemental analyzer (TC/EA; Finnigan MAT, Bremen, Germany) coupled to a Delta^plus^XP isotope ratio mass spectrometer via a ConFlo III interface (Finnigan MAT; Werner *et al*., 1999), employing the carbon reduction method of Gehre *et al*. (2004). Water samples were automatically injected into a gas chromatography PAL autosampler (CTC, Zwingen, Switzerland). To ensure consistency, we adhered to the approach ofWerner & Brand (2001) for sample and laboratory standard positioning and identical treatment during measurements. Postrun corrections (offset, memory effects, and drift) were also performed as described inWerner & Brand (2001). Long‐term quality control using lab standard water (WP‐0503‐Z0010B) yielded precisions of ±0.17‰ for δ^18^O and ±0.48‰ for δ^2^H. All samples were calibrated against the Vienna Standard Mean Ocean Water (V‐SMOW) and Standard Light Antarctic Precipitation (SLAP) for international consistency.

We used a Bayesian mixing model (Parnell *et al*., 2010) to estimate the proportional contribution of water from each soil layer to root water uptake (PC_RWU_) for each tree and campaign. We first clustered the 0–70 cm soil layers into shallow soil water (0–30 cm) and into intermediate soil water (30–70 cm). Many xylem δ^18^O and δ^2^H values were outside of the range of measured soil water samples from 0 to 70 cm depth (Fig. S3). Therefore, we used the isotopic composition of winter precipitation (i.e. precipitation isotopes from December 2021 to February 2022) as an additional end‐member for soil water deeper than 70 cm (Fig. S3). We used the Bayesian mixing model framework from the R package simmr (Govan & Parnell, 2019) to estimate the proportional contribution (PC_RWU_) from shallow (0–30 cm), intermediate (30–70 cm), and deep (> 70 cm) soil layers to xylem water of each tree (*n* = 14; beech: *n* = 6, maple: *n* = 2, fir: *n* = 3, spruce: *n* = 3) for each sampling campaign (*n* = 7). Within this framework, the posterior distribution was simulated using Markov Chain Monte Carlo simulations for which the three clustered soil layers (end members) were considered as sources and the xylem water as target. The output from the model consisted of 10 000 possible combinations of PC_RWU_ from each depth. For further analysis, we averaged PC_RWU_ per species at each sampling campaign. All analyses in the study were performed using the R software (R Core Team, 2021), with additional functionality from the dplyr and ggplot2 packages (Wickham, 2011; Wickham *et al*., 2019).

## Results

### Compound soil and atmospheric drought of 2022

On 4 August 2022, we recorded the highest air temperature (33.5°C), third‐highest vapor pressure deficit (VPD) (3.83 kPa), and the lowest soil water content (SWC) (0.11 m^3^ m^−3^ at 20 cm depth) at the Lägeren forest for the past 19 yr (2004–2022). During the 2022 summer (June, July, and August), we measured the lowest mean SWC (*c*. 0.14 m^3^ m^−3^) and the highest mean VPD (*c*. 0.95 kPa) ever recorded since 2004 (Fig. 1a). Based on these data, we identified a distinct period of compound soil and atmospheric drought (CSAD) for the mixed deciduous forest at Lägeren in the summer of 2022. The intra‐annual variability of SWC indicated an early onset of soil drought starting already in mid‐May, and further intensification in July (as shown by the shaded area in Fig. 1b,c), due to low rainfall and increasing VPD in May through July (Fig. 1b,c). A heavy rain event on 5 August 2022 (*c*. 30 mm) resulted in decreasing VPD close to normal values (long‐term mean) but in only minor recovery of soil drought. Full recovery (i.e. back to the long‐term mean) occurred only during the third week of August after multiple rain events with a cumulative rainfall of > 50 mm (Fig. 1b). Therefore, we split the summer 2022 into a pre‐CSAD period from 1 June to 13 July 2022, the CSAD period from 14 July to 4 August 2022 (ended by rain event on 5 August; Scapucci *et al*., 2024), and a post‐CSAD from 5 to 31 August 2022.

**Fig. 1:**
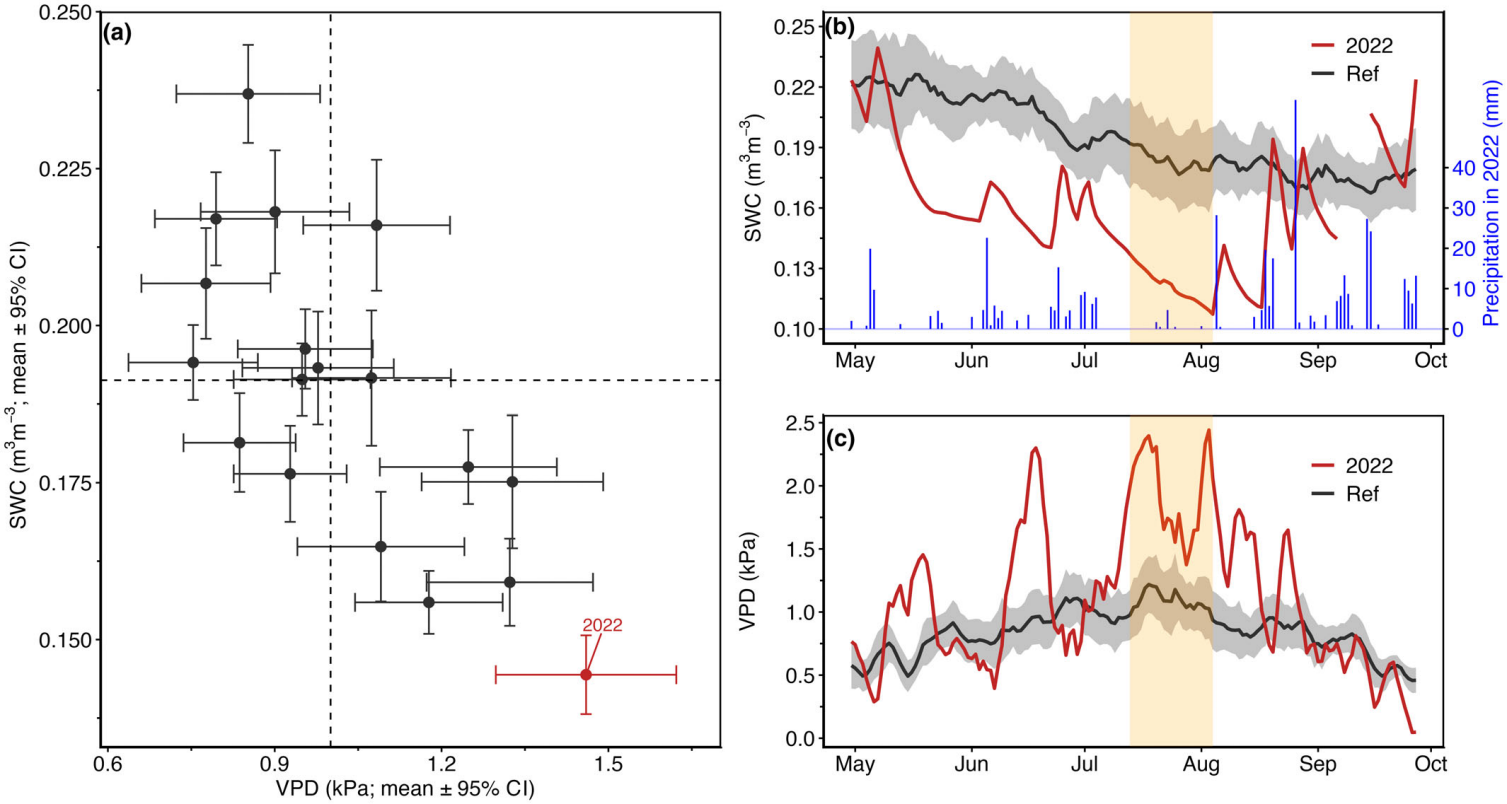
Summer of 2022 as a compound soil and atmospheric drought (CSAD). (a) Inter‐annual variability of mean soil water content (SWC, measured at 20 cm depth) and mean vapor pressure deficit (VPD) in the summer months (June, July, and August) from 2004 to 2022 (19 yr). Intra‐annual variability of (b) daily mean SWC (at 20 cm depth) and precipitation, and (c) daily mean VPD (based on a 5‐d moving average) during the 2022 growing season (red lines). The horizontal and vertical dashed lines in (a) show the mean (2004–2021) summer SWC and VPD, respectively. ‘Ref’ in (b, c) show the mean ± 95% confidence interval (CI) of SWC and VPD from 2004 to 2021. The shaded yellow areas in (b, c) show the CSAD period at the forest site (14 July to 4 August 2022).

### Ecosystem‐scale water relations

During the 2004–2021 observation period, evapotranspiration (ET) at the Lägeren forest site typically followed a distinct seasonality, with ET peaking in early July (‘Ref’ in Fig. 2a). However, the dynamics of ET in 2022 were substantially different (‘2022’ vs Ref in Fig. 2a). In 2022, ET increased in mid‐May and mid‐June (Fig. 2a) due to increased VPD (Figs 1c, 2c), despite no change or a decrease in surface canopy conductance (Gs) on some days (Fig. 2b). By contrast, during the peak drought in July, both ET and Gs significantly decreased (as much as > 50% compared with the typical long‐term mean; Fig. 2a,b) and remained low until late August. This drastic decrease in ET between July and August was mainly driven by low SWC and not by increased VPD, as shown by the consistently negative SHAP values for SWC during the 2022 CSAD (Fig. 2c), while positive SHAP values for VPD stayed relatively stable during summer 2022. Eventually, with the recovery of SWC and the decrease in VPD after mid‐August (Fig. 1b,c), both ET and Gs increased again, but with a lag of 1 wk. Full recovery (back to the long‐term mean) was reached in September (Fig. 2a–c). Gs sensitivity to VPD was significantly lower in 2022 (0.0069 ms^−1^ log_e_(kPa)^−1^) compared with the reference period (0.0168 ms^−1^ log_e_(kPa)^−1^; Fig. 2d).

**Fig. 2:**
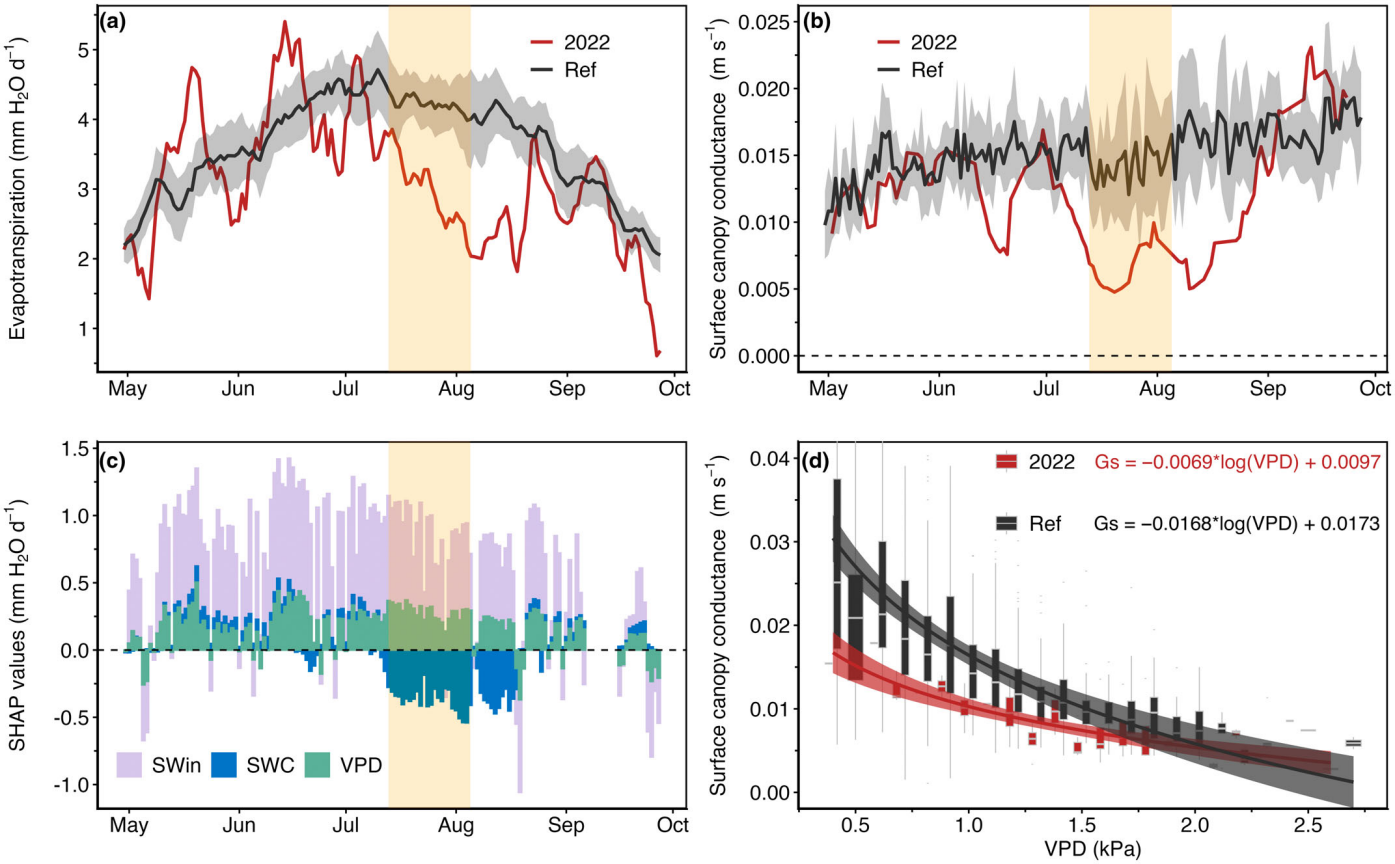
Ecosystem‐scale response of forest to 2022 compound soil and atmospheric drought (CSAD). Ecosystem‐scale response of (a) evapotranspiration, and (b) surface canopy conductance (Gs) at the Lägeren forest site in 2022 in comparison with the reference period (Ref: 2004–2021), along with the (c) drivers of evapotranspiration (SWin: short‐wave incoming radiation, soil water content (SWC), vapor pressure deficit (VPD)) in 2022 as indicated by their SHapley Additive exPlanations (SHAP) values (as stacked bars), and (d) sensitivity of Gs to VPD. Evapotranspiration and Gs in (a) and (b) represent 5‐d moving averages (centered). The shaded areas in (a, b) around the ‘Ref’ represent the 95% confidence interval (CI) of the 5‐d moving averages. In (d), Gs values are shown as box plots binned at 0.1 kPa VPD. The shaded yellow areas in (a–c) show the CSAD period at the Lägeren forest site (14 July to 4 August 2022).

### Tree‐scale water relations

#### Leaf‐level leaf water potential and stomatal conductance

At tree‐scale, we explored leaf‐, stem‐, and root‐level responses and recovery of water relations across the four tree species. During the CSAD, midday leaf water potential (LWP_md_) was *c*. 0.4 MPa lower than predawn leaf water potential (LWP_pd_; Fig. 3a,b). Between 14 July and 22 July, we observed minor variations in LWP (both at midday and predawn) even under high VPD (> 2 kPa). However, with the intensification of CSAD, we observed a significant (*P* < 0.05) decrease in both LWP_pd_ and LWP_md_ on the last and most extreme day of the CSAD (4 August), with LWP_pd_ being as low as LWP_md_ of the previous measurement days (−1.7 MPa; Fig. 3a,b). Among different tree species, beech had the lowest LWP_md_ of −2.2 MPa (on 4 August; peak intensity of 2022 CSAD) and the lowest recovery of LWP after the CSAD (Fig. 3b). In 2022, the LWPs of all tree species were significantly lower (*c*. 0.5 MPa during peak drought) than in the non‐CSAD years (2020 and 2021), indicating lower water stress in the non‐CSAD years (Fig. 3a,b). The LWP of all four tree species showed significant (*P* < 0.05) relationships with VPD in 2022, but not during the non‐CSAD years, except for beech (2020 and 2021; Fig. 3c). The LWP sensitivity to VPD did not differ significantly between broadleaf and coniferous tree species (Figs 3c, S4). However, there were contrasting effects of SWC on LWP during CSAD: significant main effects of SWC on LWP of coniferous species and nonsignificant effects for broadleaf species (Fig. 3c). During non‐CSAD years, the LWP of coniferous species showed no sensitivity to VPD and SWC (Figs 3c, S4). Nevertheless, LWP of all four tree species completely recovered to non‐CSAD LWP levels by the end of the 2022 growing season (i.e. after drought recovery in September; Fig. 3b).

**Fig. 3:**
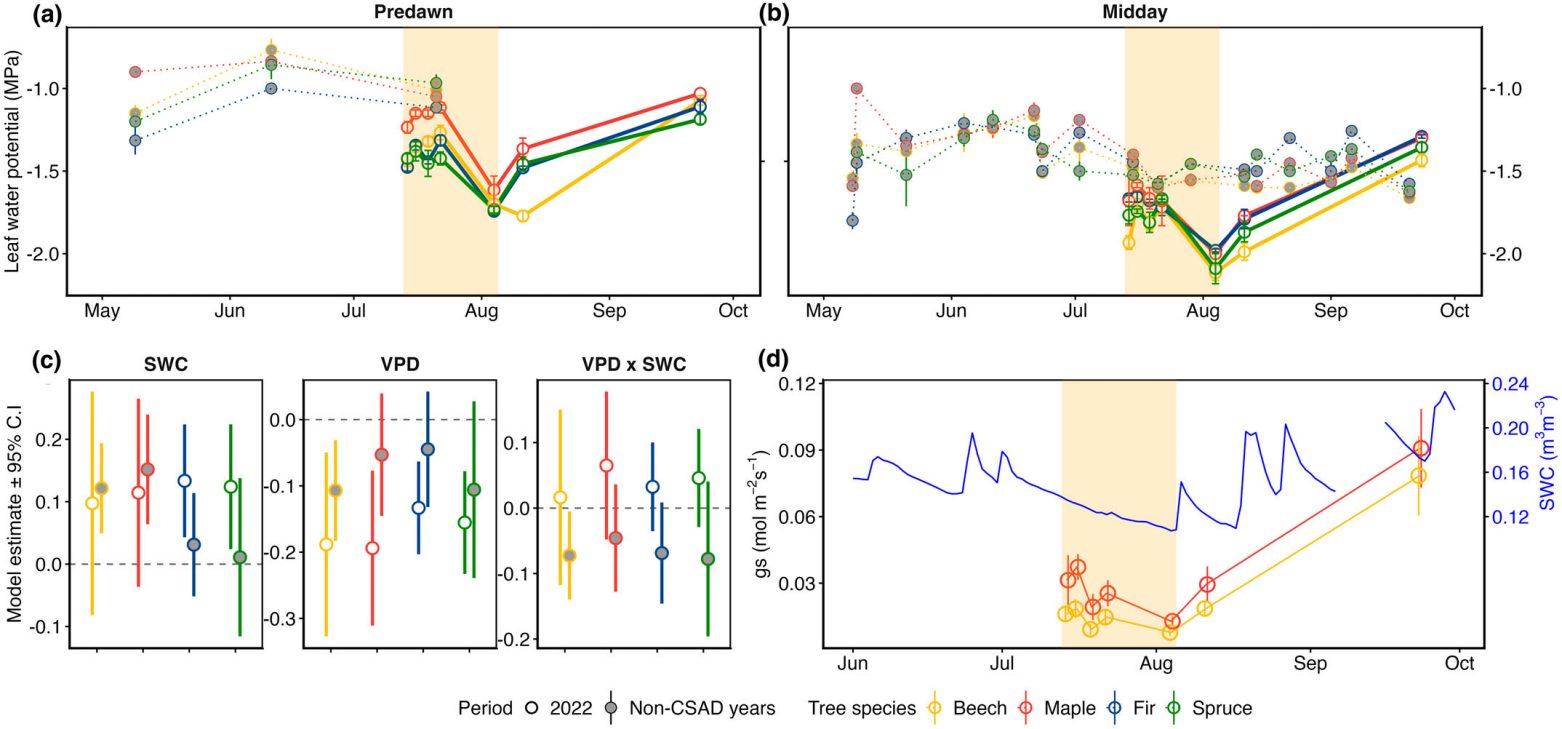
Leaf‐level response of forest to 2022 compound soil and atmospheric drought (CSAD). Leaf‐level response of (a) leaf water potentials (LWPs) measured at predawn and (b) at midday in 2022 (solid lines) and previous non‐CSAD years (2020 and 2021; dashed lines) for four tree species (beech, maple, fir, spruce), and (c) the effect of soil water content (SWC), vapor pressure deficit (VPD), and SWC × VPD (interaction) on LWP based on a linear regression model (Eqn 2). (d) Leaf stomatal conductance (gs) and soil water content (SWC; at 20 cm depth) measured in 2022 for the two broadleaf species. Leaf‐level measurements for four tree species were carried out on 7 d in 2022, with 5 d during the 2022 CSAD period (14 July to 4 August 2022) and 2 d in post‐CSAD (mid‐August and late‐September). The shaded yellow area in (a, b, and d) shows the CSAD period at the Lägeren forest site (14 July to 4 August 2022). The error bars in (a–d) represent 95% confidence interval (CI) of mean.

As expected, LWP_md_ had significant (quadratic) relationships (*R* ^2^ > 0.9; *P* < 0.01) with leaf‐level stomatal conductance (gs) for both broadleaf species (Fig. S5a). The gs of both beech and maple trees decreased as the 2022 CSAD intensified and recovered again in the post‐CSAD period (Fig. 3d). Similar to LWP (both predawn and midday), we observed lowest gs on the most intense day of the 2022 CSAD (4 August 2022; Fig. 3d). The sensitivity of leaf‐level gs to VPD and SWC was similar for both species as indicated by the similar slope of the Oren model (*m* = 0.04 ms^−1^ log_e_(kPa)^−1^; see Eqn 1) and the linear fits (Fig. S5b,c).

#### Stem‐level nighttime stem rehydration

To understand the tree‐level response and recovery of water relations, we calculated the stem rehydration ratio (SRR) in summer (JJA) of 2022, separated in three separate periods, pre‐CSAD, CSAD, and post‐CSAD (Figs S2, S6). Our results showed that during the pre‐CSAD period, SRR was greater than one for all four tree species (mean SRR = 1.3), implying that on average nighttime rehydration of xylem water was 30% larger than daytime water losses due to transpiration. Beech showed the highest SRR (mean > 2.5; Fig. 4a). However, during the CSAD period, SRR significantly decreased for all species compared with the CSAD period (Fig. S6), with contrasting SRR values for broadleaf and coniferous species. The coniferous species showed a mean SRR lower than one (mean value of 0.93, and 0.75 for fir and spruce, respectively), implying net water losses from the stem during the intense compound drought period (Figs 4b, S6). Only beech was able to maintain nighttime stem rehydration (NSR) (mean SRR ≈ 1.5) during the CSAD period, with maple just balancing out daytime dehydration and nighttime rehydration (mean SRR ≈ 1; Fig. 4b). Nevertheless, the SRR was lower than one (net dehydration) for all the tree species on the most intense day of CSAD (4 August 2022; Fig. S6). After rain events in August 2022 led to soil moisture recovery (Fig. 1b), NSR recovered completely (mean SRR > 1.3) for all tree species (Figs 4c, S6). The contrasting response of beech in comparison with other species was also evident from its nonsignificant (*P* > 0.05) SRR relationship to both nighttime VPD and SWC (at 20 cm depth; Fig. 4d,e). The SRR of maple was significantly related to SWC but not to VPD (Fig. S6). For the other two species, the SRR was significantly related to both VPD and SWC (Fig. 4d,e).

**Fig. 4:**
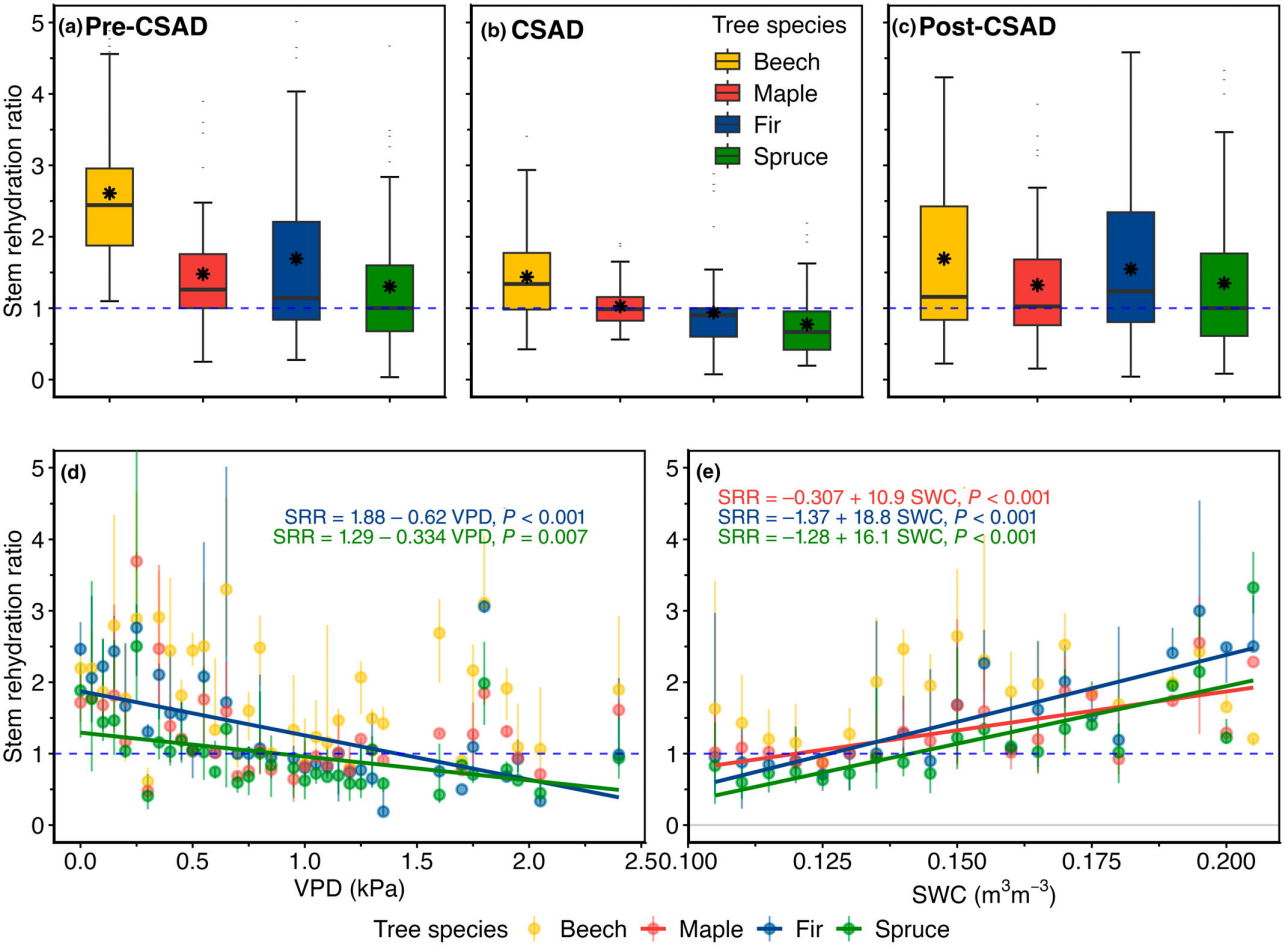
Stem‐level response of forest to 2022 compound soil and atmospheric drought (CSAD). The stem rehydration ratio (SRR) of all four tree species during (a) pre‐CSAD (1 June to 13 July 2022), (b) CSAD (14 July to 4 August 2022), and (c) post‐CSAD (5 August to 31 August 2022). Relationships of SRR (median) with (d) nighttime vapor pressure deficit (VPD) and (e) nighttime soil water content (SWC) at 20 cm soil depth for all four tree species (beech, maple, fir, and spruce) during the summer (JJA) of 2022. The upper and lower limit of the box in the boxplots (a–c) show first and third quartile. The asterisks inside the boxplots (a‐c) show the mean value. The error bars (in d, e) represent the 25^th^ and 75^th^ percentile values of SRRs. Nonsignificant (*P* > 0.05) relationships in ‘d’ and ‘e’ are not shown. The SRR = 1 (represented by horizontal, dashed blue line), above 1, and below 1 indicate 100%, > 100%, and < 100% restoration of nighttime xylem water storage compared with the daytime stem water losses due to transpiration, respectively. See Supporting Information Fig. S2 for estimation of SRR.

#### Root‐level root water uptake depths

With some exceptions, soil water isotope profiles showed the expected depth profiles, with higher isotope ratios at shallow depths compared with deeper depths (Figs 5a, S7a). As the CSAD intensified, both soil and xylem water became isotopically lighter (more negative δ^2^H and δ^18^O values), followed by becoming more enriched during post‐CSAD due to more enriched isotopic signatures in summer rainfall (Figs 5a, S7a). The proportional contributions to root water uptake (PC_RWU_), estimated based on δ^18^O and δ^2^H, added up very well in the mixing model calculations (uncertainties smaller than 10%; Fig. S8) among the three soil layers (shallow, intermediate, deep), with a more pronounced pattern in δ^2^H‐based PC_RWU_ than δ^18^O‐based PC_RWU_ (Figs 5b–d, S7b–d). During the CSAD, our results showed contrasting patterns of RWU depth for broadleaf (beech and maple) and coniferous (fir and spruce) tree species. Broadleaf species showed higher PC_RWU_ from deep soil layers (i.e. winter precipitation stored below 70 cm depth) compared with those of the coniferous species (35% to 50% vs < 25%; Fig. 5d; see Fig. S9 for the PC_RWU_ distribution). Coniferous trees sourced their water largely from shallower soil layers (0–30 cm), followed by intermediate (30–70 cm) and only little water from the deep layers: PC_RWU_ of fir and spruce remained largely unchanged, *c*. 45%, 30%, and 25% from shallow, intermediate, and deep soil layers, respectively (Fig. 5b), even as the CSAD intensified (from 14 June to 4 August 2022). Among the two broadleaf species, beech showed sourcing of deep soil water, with *c*. 50% PC_RWU_ from the deepest layer during the CSAD (Fig. 5d). By the end of the growing season and well beyond the CSAD, that is 29 September 2022, all four tree species largely sourced water from shallow and intermediate soil layers, with only 20–25% of root water uptake (RWU) originating from the deep soil layer.

**Fig. 5:**
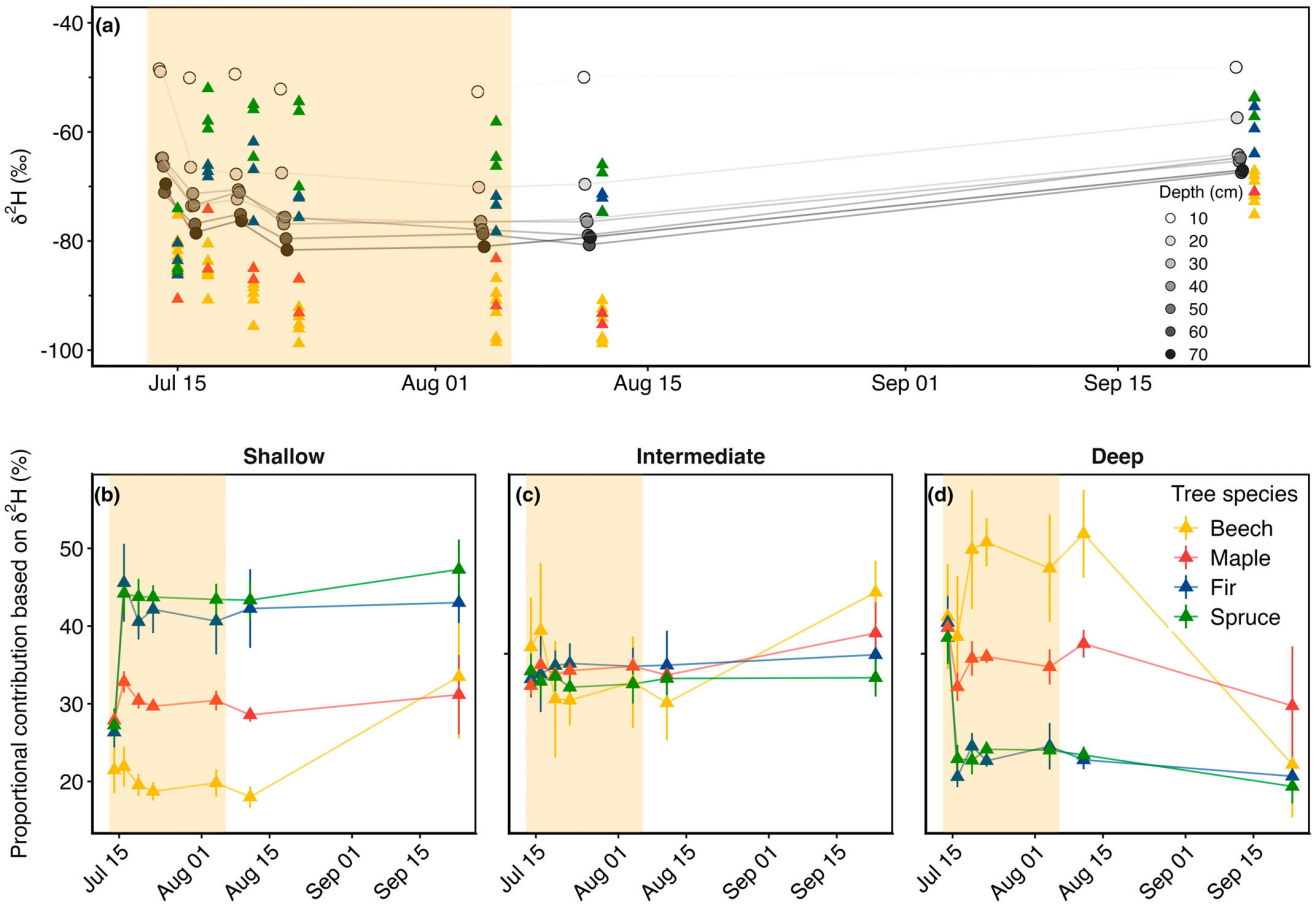
Root‐level response of forest to 2022 compound soil and atmospheric drought (CSAD). (a) Temporal variations of δ^2^H in soil water from different depths (circles), and xylem water from four tree species (in triangles; beech, maple, fir, and spruce). Soil water was extracted from bulk soil sampled at 0–10, 10–20, 20–30, 30–40, 40–50, 50–60, and 60–70 cm depth, with the lower depth being used for the legends. Proportional contributions to root water uptake (PC_RWU_) of (b) shallow (0–30 cm), (c) intermediate (30–70 cm) and (d) deep (> 70 cm) soil layers estimated based on δ^2^H for four tree species using a Bayesian mixing model (see Supporting Information Fig. S8 for the distribution of PC_RWU_). Means and errors (±95% confidence interval (CI)) are given in (b–d). The shaded yellow areas show the CSAD period at the Lägeren forest site (14 July to 4 August 2022).

## Discussion

The summer of 2022 was characterized by compound soil and atmospheric drought (CSAD) conditions at the Lägeren mixed deciduous forest site (CH‐Lae), providing a unique opportunity to examine the link between ecosystem‐, tree‐, and leaf‐level responses and recovery mechanisms. The results showed that ecosystem‐scale responses during the 2022 CSAD were closely tied to tree‐scale ecophysiology, highlighting a strong connection between canopy processes and individual tree‐species water use strategies (Fig. 6). All three hypotheses were largely supported by our results, with ecosystem‐scale evapotranspiration (ET) being directly related to leaf‐level leaf water potential (LWP). The relationships of LWP with stem‐level stem nighttime rehydration varied across species, which in turn were directly related to species‐specific root‐level root water uptake (RWU) depths.

**Fig. 6:**
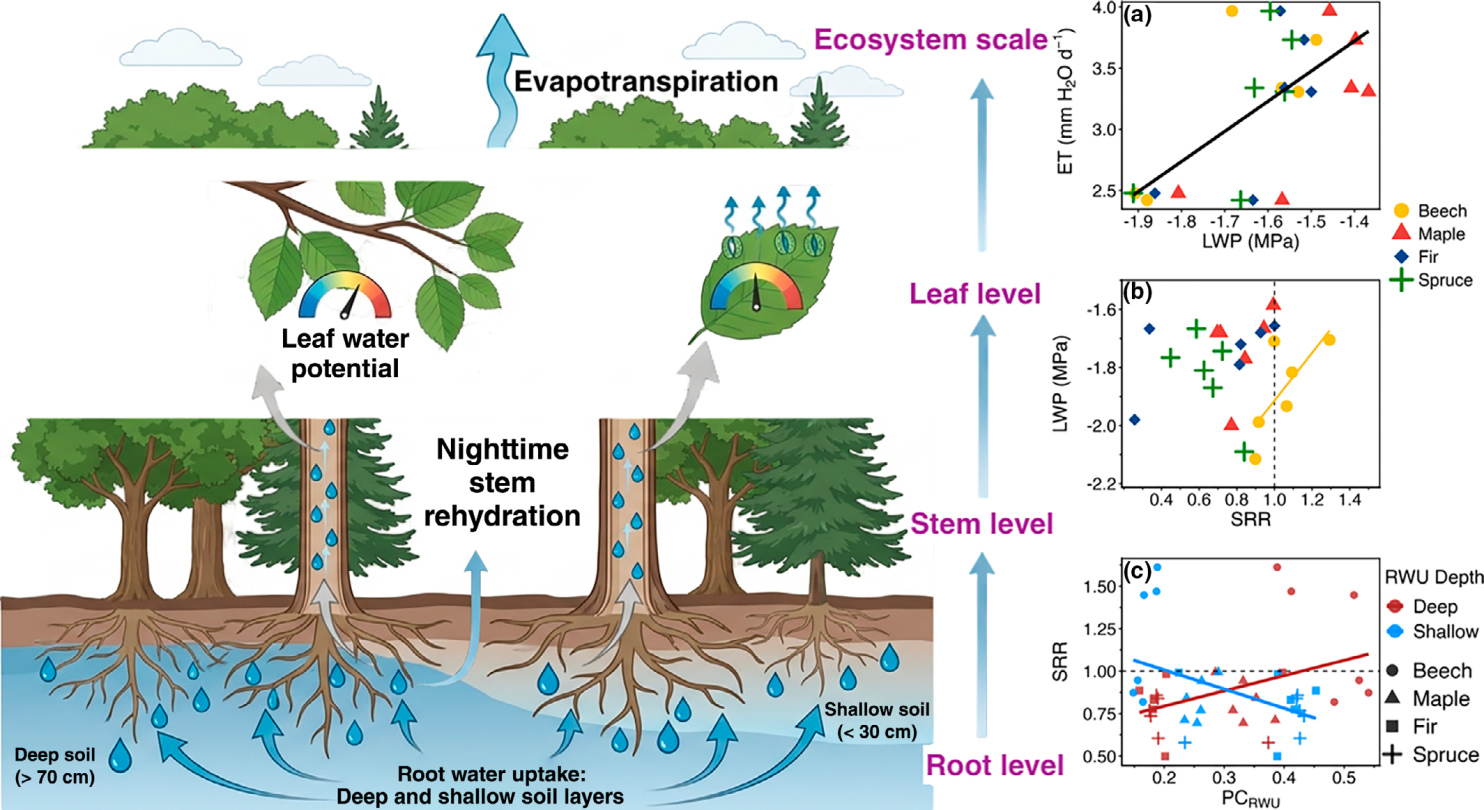
Schematic showing the links of tree‐scale (root‐, stem‐, and leaf‐level) and ecosystem‐scale water relations during the compound soil and atmospheric drought (CSAD) at CH‐Lae. (a) Reduced ET is linked to leaf‐level physiological responses, that is to a decrease in leaf water potential (LWP). (b) The reduction in LWP is not clearly related to nighttime stem rehydration for all species (maple, fir, and spruce), but for beech, a lower stem rehydration ratio (SRR) results in lower LWP. (c) The SRR is directly related to a proportional contribution of deep root water uptake (PC_RWU_) depth, indicating that with increasing contributions of deep RWU depth, SRR increases. The curved arrows show the transport of water. The figure was created using Google Gemini 3 Pro Image model (last accessed June 2026) to create an initial draft and was later modified in the Apple Preview software v.11.0 (1113.5.3). All contents were reviewed and approved by the co‐authors.

### Ecosystem‐scale water relations

The pre‐CSAD period (1 June to 13 July 2022) was characterized by high vapor pressure deficit (VPD) (daily mean of 1.75 kPa) and declining but not limiting soil water content (SWC), resulting in moderately dry conditions. During such conditions, high atmospheric water demand typically increases ET (Diao *et al*., 2021; He *et al*., 2022; Shekhar *et al*., 2024b), which accelerates soil water depletion as observed in this study. Furthermore, during the pre‐CSAD period, surface canopy conductance (Gs) was similar to the long‐term mean, clearly indicating VPD control of forest ET (Novick *et al*., 2016). However, with further decreasing SWC in July 2022, both ET and Gs showed a substantial decrease due to limited soil water supply to the trees (Fig. 2c), supporting earlier studies (Miralles *et al*., 2019; Ahmed *et al*., 2021). The reduction in ET during the CSAD period was most likely due to decreased tree transpiration, as supported by the observation that *c*. 90% of ET during summer is water loss due to transpiration (Paul‐Limoges *et al*., 2020). This ecosystem‐scale regulation in ET and Gs during CSAD was directly dependent on the leaf‐level physiological responses of reduced LWP (Fig. 6a) and stomatal conductance (gs), thereby supporting our Hypothesis 1. The effect of decreased soil evaporation during the CSAD period due to soil moisture limitations is probably minor, since the forest canopy at Lägeren is quite dense (leaf area index of *c*. 4; Ruehr & Buchmann, 2010), and soil evaporation is typically low when leaf area index is high (Schulze *et al*., 1994). In addition to increasing VPD, the reduced soil moisture availability during the CSAD also limited Gs, as trees closed stomata in order to prevent hydraulic failure, as seen in measured gs and LWP and reported earlier (Buckley, 2005; Grossiord *et al*., 2020; Shekhar *et al*., 2024b).

However, even after the end of the CSAD period, following a short and heavy precipitation event (*c*. 26 mm in 2 h) on 5 August 2022, ET continued to be significantly lower than the long‐term mean as soil drought conditions persisted (Fig. 1b). Thus, short and heavy precipitation during extreme soil dryness periods, as observed during the 2022 CSAD, still resulted in only limited soil water recharge (Fowler *et al*., 2021). Reduced ET (and the accompanying limited atmospheric humidity) during CSAD lead to an altered energy balance, ultimately resulting in increased sensible heat fluxes, further intensifying the CSAD event, as also seen for other forests (Miralles *et al*., 2019; Graf *et al*., 2020). With multiple precipitation events during the third week of August 2022 (post‐CSAD), SWC at the Lägeren forest site fully recovered. The ecosystem‐scale response of water fluxes (i.e. ET and Gs), however, only recovered after a lag time of *c*. 1 wk. This delayed response of recovery highlights the lag between the replenishment of soil moisture and the recovery of canopy physiological functioning. The response and recovery of ET and Gs at the Lägeren forest site indicated strong limitations from soil drought during the CSAD period, as clearly identified by our SHAP analysis. This highlights the importance of site‐specific environmental conditions when characterizing CSAD effects on ecosystem water fluxes (Novick *et al*., 2016; Diao *et al*., 2021).

### Leaf‐stem‐level coordination and species‐specific hydraulic strategies

The leaf‐level stomatal regulation (gs) and LWP exhibited species‐specific behaviors that affected different drought responses. Similarly, the nighttime stem rehydration (NSR) also showed species‐specific behaviors. Our results indicated that the link between maintenance of leaf‐level gs and LWP to NSR is dependent on species‐specific hydraulic strategies, thereby supporting our second hypothesis. The predawn and midday LWP of all four tree species (beech, maple, fir, and spruce) in 2022 were lower than in non‐CSAD years, clearly highlighting stress conditions in the leaf water status (Ryan, 2011; Berdanier & Clark, 2018). Furthermore, both LWP and stem rehydration ratio (SRR) significantly declined on the most intense day of the CSAD (which was the last day of the CSAD period, 4 August 2022), with all species showing midday LWP < −1.9 MPa and SRR < 1. Species‐specific responses were clearly shown by the divergent effect of soil drought on LWP of broadleaf species (nonsignificant) and coniferous species (significant). The two coniferous tree species (spruce and fir) showed a fairly conservative stomatal regulation, that is reducing gs to limit transpiration loss and preserve LWP at higher values, thereby preventing excessive declines in LWP (Figs 3, 4), supporting earlier studies for spruce (Berdanier & Clark, 2018; Martinetti *et al*., 2025). Such conservative leaf‐level regulation of coniferous species was supported by their weak ability to recharge their stem water at night (Fig. 6b). Among the four species, beech showed the lowest LWP (*c*. −2.2 MPa) and also the slowest recovery of LWP post‐CSAD, and showed direct relationship with its NSR during CSAD (Fig. 6b). Beech is characterized by more opportunistic stomatal regulation (Leuschner *et al*., 2022), that is keeping stomata more open during extreme drought for transpiration than coniferous trees (Walthert *et al*., 2024). Furthermore, broadleaf species (beech and maple) occupied a more permissive end of the hydraulic spectrum, maintaining higher gs and sustained NSR throughout drought periods (Peters *et al*., 2023). Specifically, European beech maintained a SRR of *c*. 1.5 during the CSAD, implying that its nighttime refilling significantly exceeded its daytime transpirational losses. This robust NSR served as a critical internal buffer, preventing LWP from reaching critical cavitation thresholds and allowing for continued gas exchange (Arend *et al*., 2021). The lowest LWP measured (−2.2 MPa) for beech did not exceed its reported water potential at the turgor loss point in a drought year (−2.48 ± 0.01 MPa in 2018; Weithmann *et al*., 2022) and its reported P50 value (xylem water potential at 50% loss of hydraulic conductance of −2.8 to −3.8 MPa; Schuldt *et al*., 2016; Stojnić *et al*., 2018). This indicated that no hydraulic failure had occurred, which during the post‐CSAD period allowed slow but complete LWP recovery in beech. Nevertheless, recent studies have highlighted high vulnerability of beech trees to the 2018 drought (which also showed CSAD characteristics; Scapucci *et al*., 2024), characterized by loss of hydraulic conductance (Arend *et al*., 2022), widespread premature leaf senescence and tree mortality (Obladen *et al*., 2021; Frei *et al*., 2022). However, such extreme effects on beech trees, or any other tree species, were not observed in our study during the CSAD in 2022. It is worthwhile to mention that LWP at the higher parts of the canopy would be lower than the LWP we measured at a canopy height of 8–10 m. Thus, the top canopy foliage might have been more stressed than observed in the lower canopy.

### Stem‐Root rehydration linkage

Our results clearly supported our third hypothesis since greater SRR was related to deep water root access (Fig. 6c). Among the four studied tree species, beech and maple demonstrated the ability to access deeper soil water during the CSAD period, also seen in a previous study at the Lägeren site (Brinkmann *et al*., 2019). This ability of shifting root water uptake (RWU) depths resulted in a steady (but potentially reduced) soil water supply (see also Martinetti *et al*., 2025), which resulted in sustained NSR during the CSAD, thereby resulting in only a minimal reduction in their transpiration rate, as seen for beech at the Lägeren (Brinkmann *et al*., 2016; Peters *et al*., 2023) and also at other forest sites (Betsch *et al*., 2011; Leuschner, 2020). RWU of the other tree species (fir and spruce) originated dominantly from shallow soil layers, leading to a depletion of internal stem water storage (daytime transpiration water losses exceeding NSR) during the CSAD, which most likely resulted in substantial reductions in their transpiration rates (Ryan, 2011; Berdanier & Clark, 2018). This deep soil water access of beech compared with the conifers might be explained by more fine roots of the beech in deep soil layers compared with conifers (Walthert *et al*., 2024), likely due to genetics and/or competition effects in our mixed forest. Thus, beech and maple, with their access to deep water, were most likely responsible for the partial maintenance of the ecosystem‐scale ET at the Lägeren site.

In summary, responses and recovery of ecosystem‐scale water relations were largely related to the aggregated effects of different tree‐species‐specific water use strategies. By the end of the 2022 growing season, both LWP and gs of all trees fully recovered. The RWU depth of both beech and maple during post‐CSAD also indicated a shift back to shallower soil water sources compared with those during the CSAD period (however, still from deeper than 60 cm depth). With soil water recovery during post‐CSAD, stem rehydration also increased. The complete recovery of all studied ecosystem‐scale, tree‐scale water relations of CH‐Lae showed no short‐term (sub‐seasonal) legacy effect, highlighting the forest's resilience to CSAD. However, with increasing intensity of CSAD in future climate scenarios (Shekhar *et al*., 2024a), trees at the Lägeren forest might be pushed to or even beyond their (hydraulic) limits, potentially being unable to recover from CSAD events. Therefore, it is important to develop effective management approaches and long‐term strategies considering site‐specific ecophysiological and environmental factors influencing forest resilience to future compound extreme events (Wang *et al*., 2023).

### Conclusions

Compound soil and atmospheric drought (CSAD) periods in Europe are projected to become more frequent and intense. This study highlighted the interacting responses of ecosystem‐, tree‐, and leaf‐level water relations to the 2022 CSAD in a temperate mixed deciduous forest. The findings emphasized the role of species‐specific tree hydraulic strategies in affecting ecosystem‐scale processes, such as evapotranspiration (ET) and canopy conductance. Broadleaf species, like beech and maple, had a more drought‐tolerant strategy, enabling a shift of root water uptake (RWU) to deeper layers, while coniferous species, such as spruce and fir, had a more conservative strategy that likely restricted transpiration when soil moisture was depleted in the shallow layers. These insights underline the importance of species composition in determining forest responses to drought and recovery trajectories. Moving forward, these results provide a mechanistic foundation for assessing forest vulnerability under future climate scenarios. The findings also highlight the need for site‐specific management strategies that promote higher tree diversity, preferentially with drought‐resilient tree species and that maintain forest water relations. Future research should integrate multi‐scale modeling to predict long‐term forest responses and evaluate adaptive strategies that mitigate the effects of future CSAD on water relations in forested ecosystems.

## Competing interests

None declared.

## Author contributions

NB conceived the idea. AS, AKG and NB designed the study. AS, LS, SA‐B, AB, YW, FR, FT, RG, LK, IF, RAW and NB conducted the fieldwork and sampling. AS and AA performed the laboratory analysis. RZ and SE provided the dendrometer data. AS and LH processed the eddy covariance data. AS conducted the data analysis and wrote the first draft. AS, AKG, AA, LS, SA‐B, AB, LH, YW, FR, FT, RG, LK, IF, RAW, RZ, SE, MF, SO, MG and NB contributed substantially to the reviewing, editing, and revising the manuscript.

## Disclaimer

The New Phytologist Foundation remains neutral with regard to jurisdictional claims in maps and in any institutional affiliations.

## Supporting information


**Fig. S1** Soil water content (half‐hourly) from two different sensors at the Lägeren forest site shown in two different colors, standardized (z‐score) SWC data from two different sensors, corrected SWC for the entire study period (see ‘Ecosystem‐scale measurements’ in the Materials and Methods section).
**Fig. S2** Temporal variation of stem radius constituting reversible daytime stem dehydration and nighttime stem rehydration, which was used to calculate the stem rehydration ratio.
**Fig. S3** Water isotopes (δ^18^O, δ^2^H) in soil samples (collected from 0 to 70 cm depth), twig xylem samples of four tree species (beech, maple, fir, and spruce), and winter precipitation (December 2021 to February 2022, mean ± SD).
**Fig. S4** Relationships of leaf water potential with vapor pressure deficit, and soil water content for all four tree species (beech, maple, fir, and spruce) during the non‐CSAD years (2020 and 2021), and the CSAD year of 2022.
**Fig. S5** Relationships of leaf (beech and maple) stomatal conductance with midday leaf water potential, vapor pressure deficit, and soil water content at 20 cm depth.
**Fig. S6** Variations of stem rehydration ratio during the summer months for all four tree species at the Lägeren forest site.
**Fig. S7** Temporal variations of δ^18^O of soil water from different depths, and xylem water from four tree species (beech, maple, fir, spruce), and proportional contributions to root water uptake (PC_RWU_) of shallow (0–30 cm), intermediate (30–70 cm) and deep (> 70 cm) soil layers estimated based on δ^2^H for four tree species using a Bayesian mixing model.
**Fig. S8** Comparison of the proportional contributions to root water uptake (PC_RWU_) estimated based on δ^18^O and δ^2^H in shallow (0–30 cm), intermediate (30–70 cm) and deep (> 70 cm) soil layers for 14 trees of four tree species (beech, maple, fir, and spruce) over all the 7 d of this study.
**Fig. S9** Frequency density plots of proportional contribution to root water uptake (PC_RWU_) estimated based on δ^2^H and δ^18^O in shallow (0–30 cm), intermediate (30–70 cm) and deep (> 70 cm) soil layers for 14 trees of four tree species (beech, maple, fir, and spruce) over all the 7 d of measurement.
Please note: Wiley is not responsible for the content or functionality of any Supporting Information supplied by the authors. Any queries (other than missing material) should be directed to the *New Phytologist* Central Office.

## Data Availability

The eddy covariance‐based ET data are available at ETH Research Collection (doi: 10.3929/ethz‐b‐000582198; Hörtnagl *et al*., 2023). The R scripts used for the data analyses and plots are available upon request from the corresponding author. The tree‐level datasets used are available at the ETH Research Collection (doi: 10.3929/ethz‐c‐000803593; Shekhar *et al*., 2026).
