# Supplementary material for: Forest under stress: responses and recovery of water relations across scales during the compound drought in summer 2022
Source: New Phytol. 2026 Sep 4;252(3):1050–64. doi: 10.1111/nph.71516 (PMC13628538; doi:10.1111/nph.71516)
Supplement: Supplementary file 1 [file NPH-252-1050-s001.pdf]

New Phytologist Supporting Information

Article title: Forest under stress: responses and recovery of water relations across scales during the compound drought in summer 2022

Authors: Ankit Shekhar[1*], Anna K. Gilgen[2], Annika Ackermann[2], Liliana Scapucci[2], Sergio Aranda-Barranco[3], Anastasiia Bolshakova[4], Lukas Hörtnagl[2], Yi Wang[2,5], Franziska Richter[2,6], Fabio Turco[2], Ruikun Gou[7], Luana Krebs[2,8], Iris Feigenwinter[2], Roland A. Werner[2], Roman Zweifel[6], Sophia Etzold[7], Marius Floriancic[2,9], Stefan Osterwalder[2,10], Mana Gharun[11], Nina Buchmann[2]

[1] Agricultural and Food Engineering Department, IIT Kharagpur, 721302 Kharagpur, India
[2] Institute of Agricultural Sciences, ETH Zurich, 8092 Zurich, Switzerland
[3] Andalusian Institute for Earth System Research, University of Granada, 18006 Granada, Spain
[4] Department of Civil Engineering, Lassonde School of Engineering, York University, M3J 1P3 Toronto, Canada
[5] ICOS ERIC Carbon Portal, Department of Earth and Environmental Sciences, Lund University, 22362 Lund, Sweden
[6] Swiss Federal Institute for Forest, Snow and Landscape Research (WSL), 8903 Birmensdorf, Switzerland
[7] College of Forestry, Northwest A&F University, Yangling, 712100, China
[8] Department of Earth System Science, Stanford University, Stanford, 94305 CA, USA
[9] Department of Civil, Environmental and Geomatic Engineering ETH Zurich, 8903 Zurich, Switzerland
[10] Department of Geography, University of Zurich, 8057 Zurich, Switzerland
[11] Faculty of Geosciences, University of Münster, 48149 Münster, Germany
* Correspondence to Ankit Shekhar (ankit.shekhar@agfe.iitkgp.ac.in)

Article acceptance data: 20 July 2026

The following Supporting Information is available for this article:

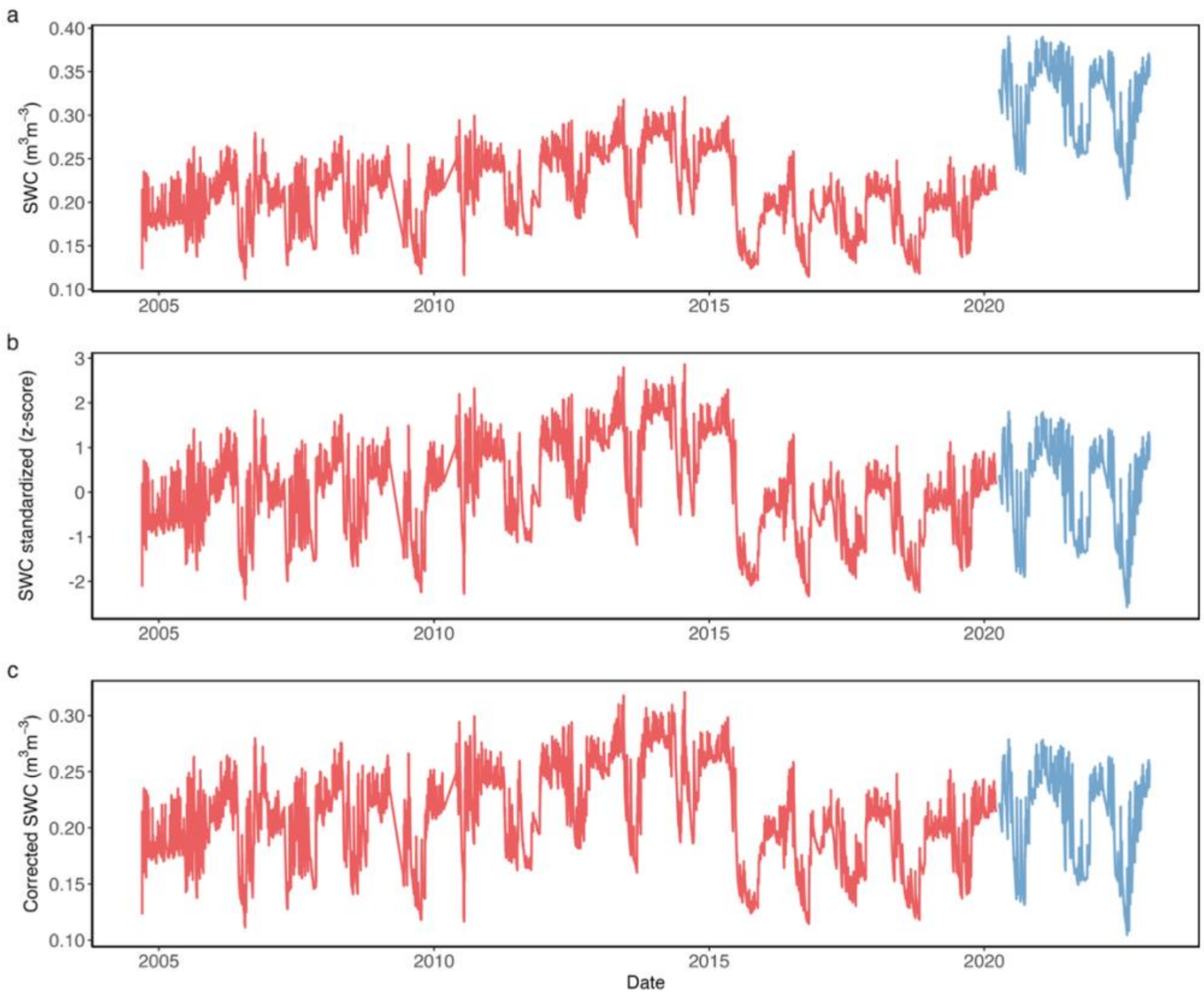

Figure S1. Soil water content (half-hourly) from two different sensors at the Lägeren forest site (a) shown in two different colors, (b) standardized (z-score) SWC data from two different sensors, (c) corrected SWC for the entire study period (see Section 2.2).

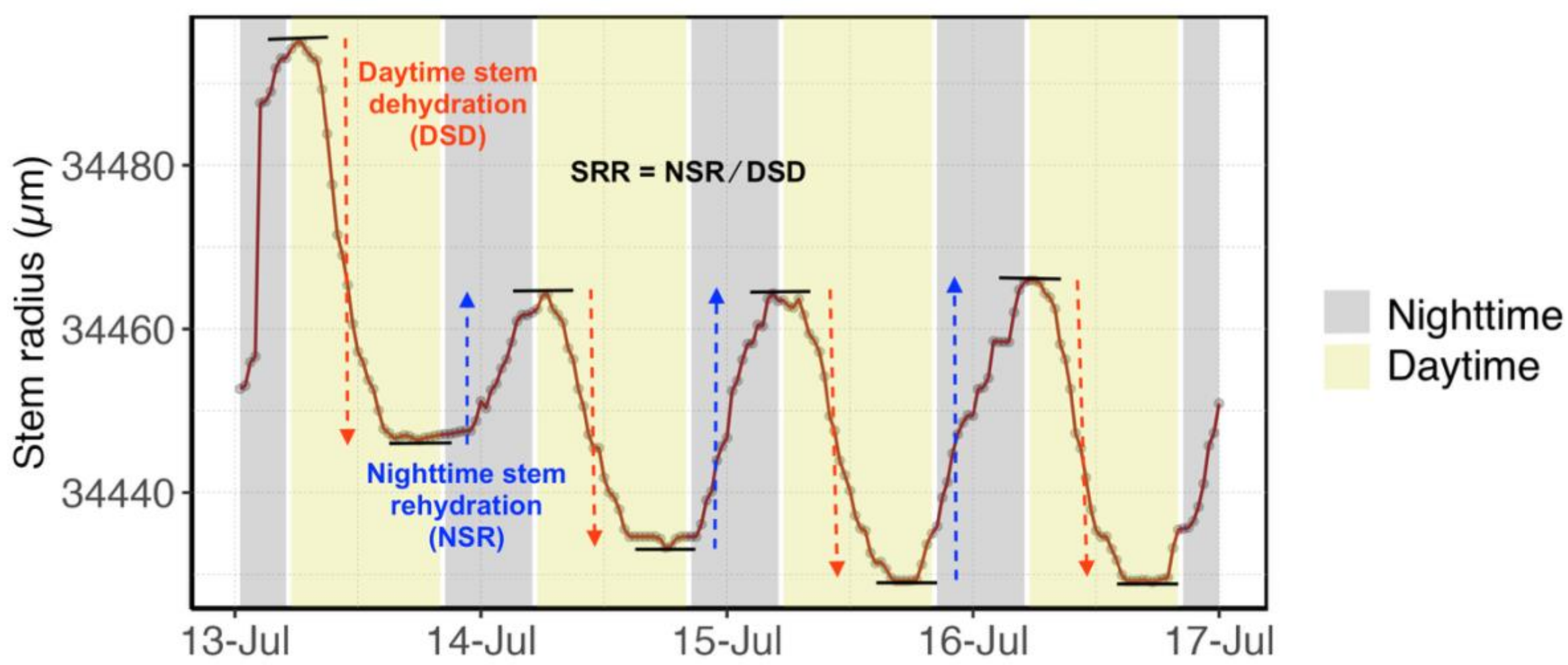

Figure S2. Temporal variation of stem radius constituting reversible daytime stem dehydration (DSD) and nighttime stem rehydration (NSR), which was used to calculate the stem rehydration ratio (SRR). Night- and daytime are shown as well. Data shown as an example are from a fir tree.

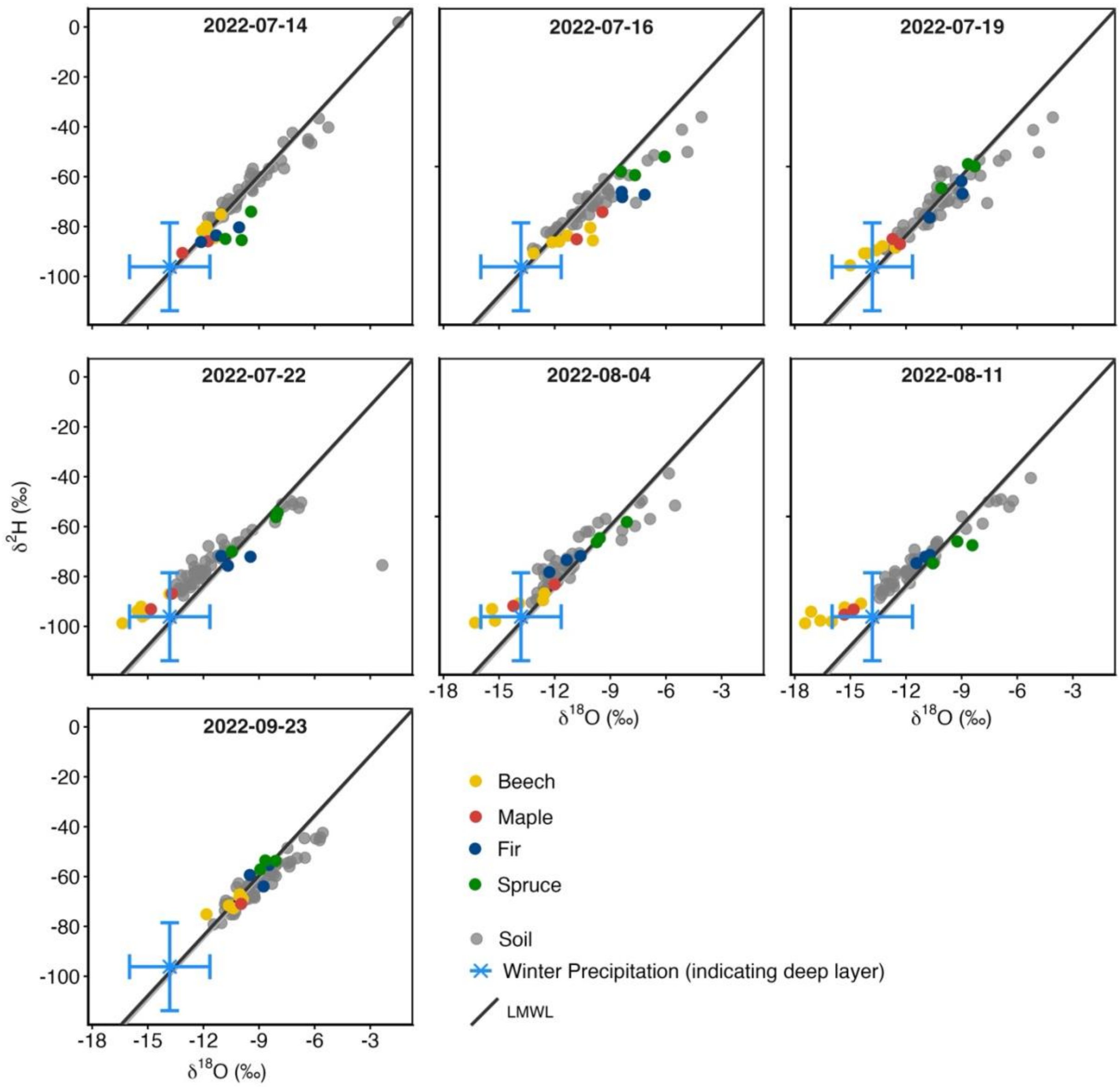

Figure S3. Water isotopes (δ^{18}O, δ^{2}H) in soil samples (collected from 0-70 cm depth), twig xylem samples of four tree species (beech, maple, fir, spruce), and winter precipitation (December 2021 to February 2022, mean ± S.D.). Winter precipitation was used in this study to represent the deep soil layer (> 70 cm) in the Bayesian mixing model. The black line shows the local meteoric water line (LMWL) of the Lägeren site (obtained from Brinkmann et al., 2019).

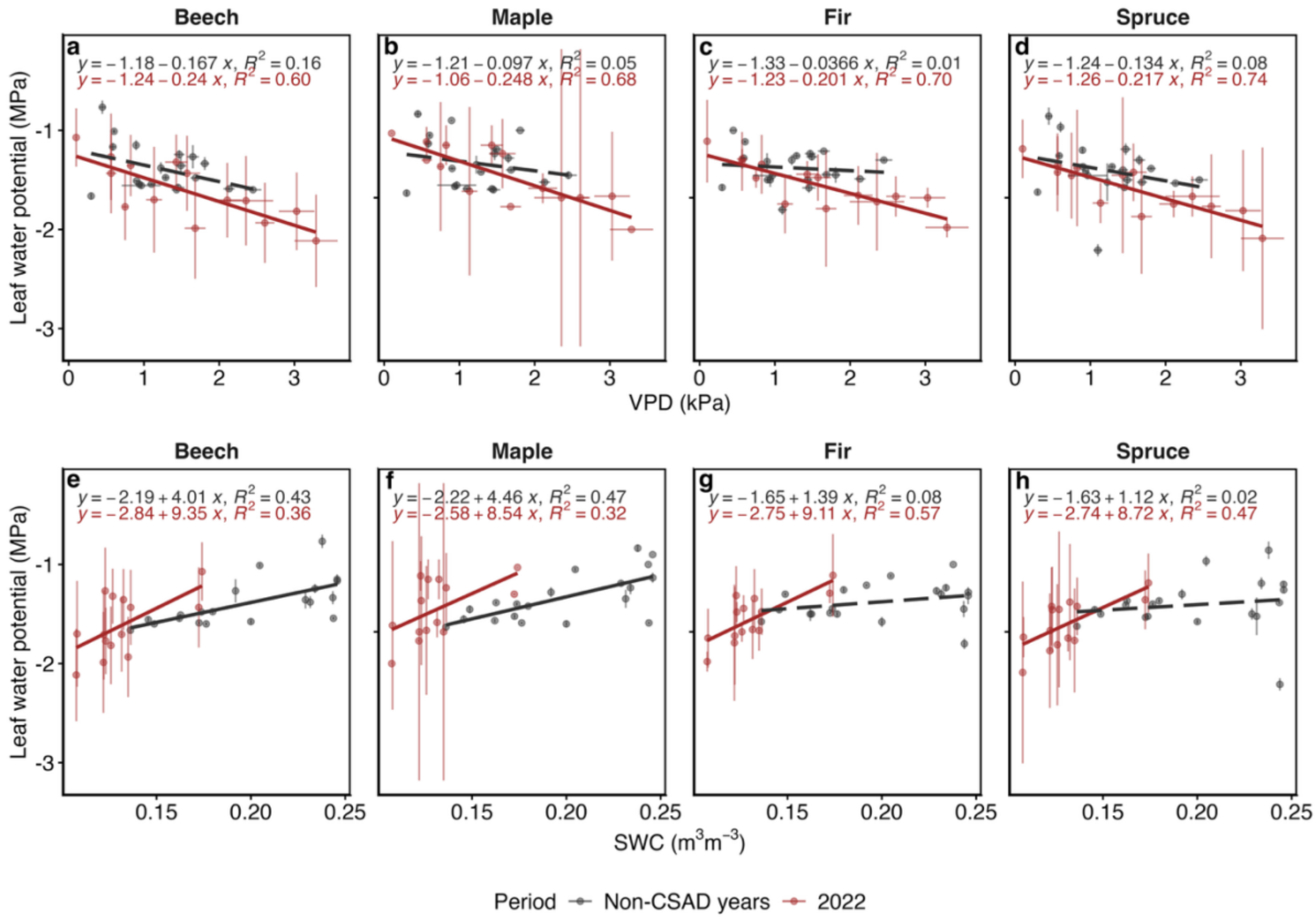

Figure S4. Relationships of leaf water potential with (a-d) vapor pressure deficit (VPD), and (e-h) soil water content (SWC) for all four tree species (beech, maple, fir, spruce) during the non-CSAD years (2020 and 2021 in black), and the CSAD year of 2022 (red). Mean ± se are plotted. The significant ($p<0.05$) and non-significant relationships are indicated by solid and dashed lines, respectively.

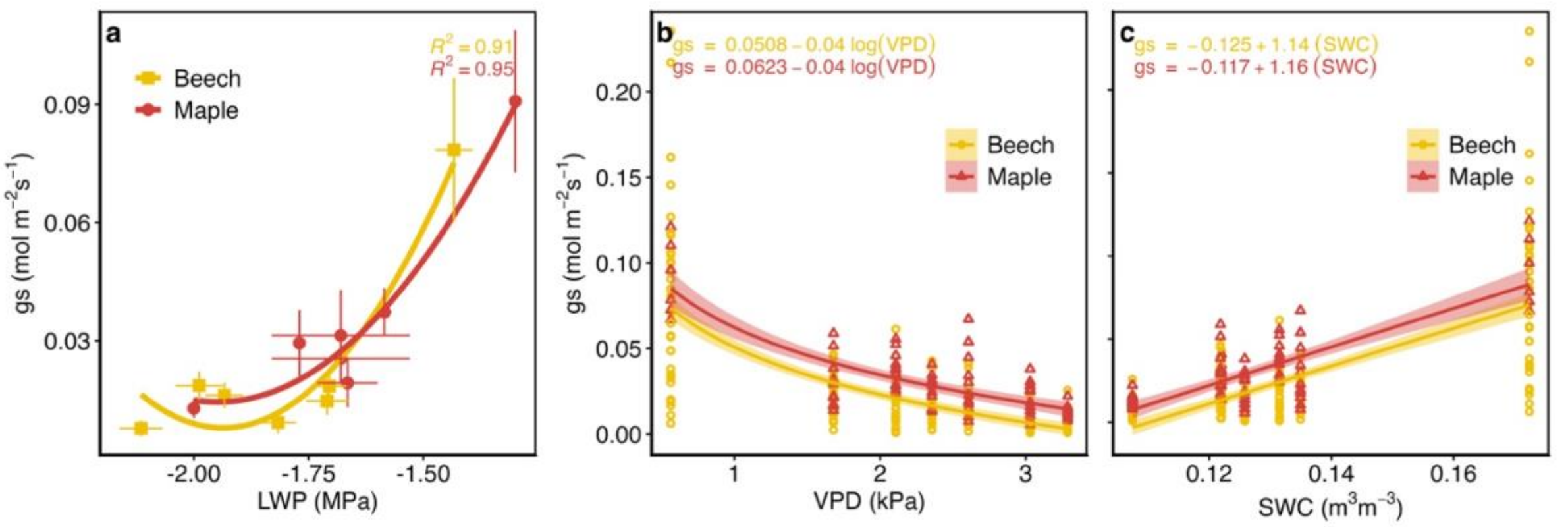

Figure S5. Relationships of leaf (beech and maple) stomatal conductance (gs) with (a) midday leaf water potential (LWP), (b) vapor pressure deficit (VPD), and (c) soil water content (SWC) at 20 cm depth. All three relationships are significant ($p<0.05$) and similar for beech and maple.

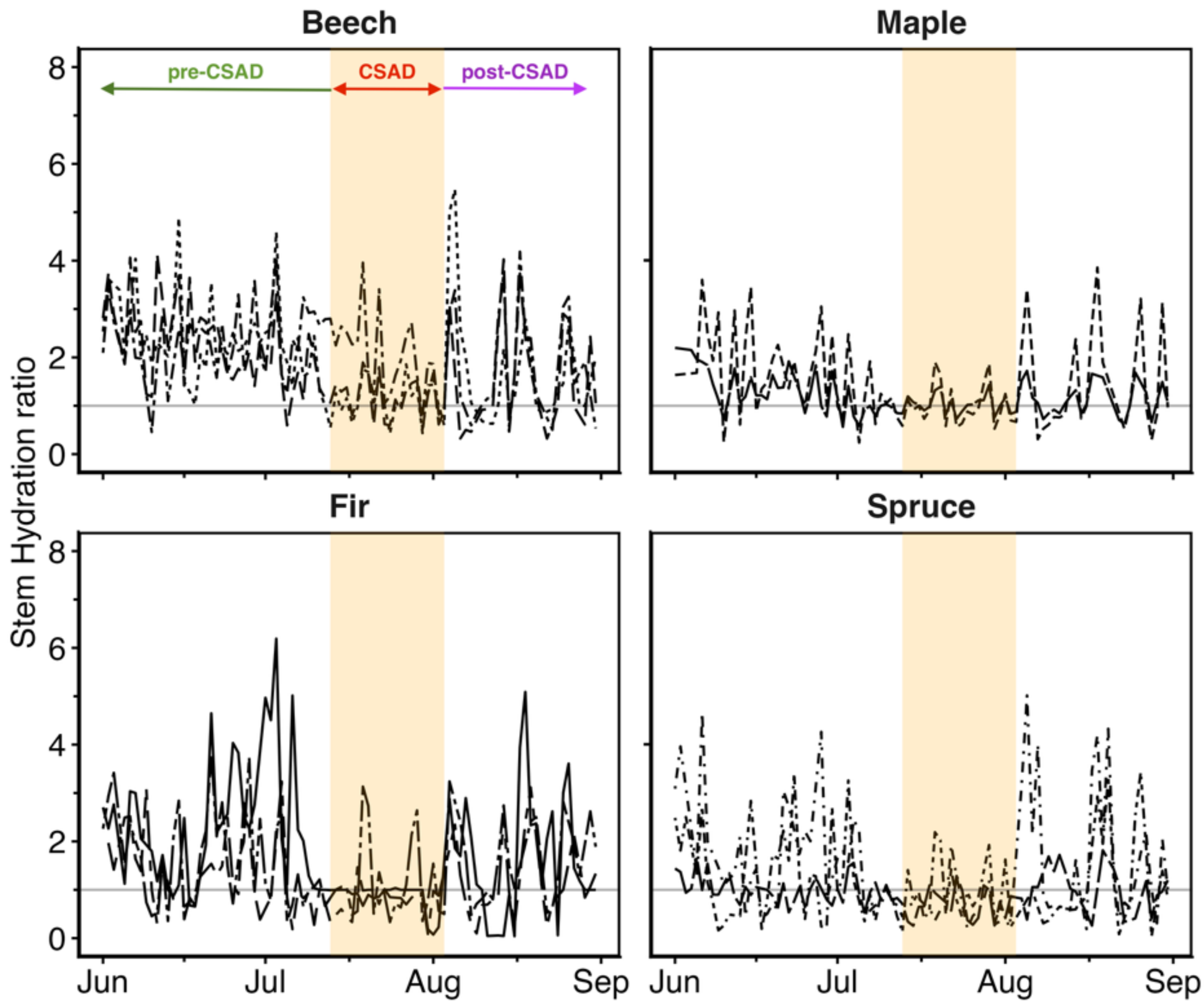

Figure S6. Variations of stem rehydration ratio (SRR) during the summer months (JJA) for all four tree species at the Lägeren forest site. Summer 2022 is divided into pre-CSAD (1st June to 13th July 2022), CSAD (14th July to 4th August 2022; shaded areas), and post-CSAD (5th August to 31st August 2022). SRR > 1 implies a net rehydration, whereas SRR < 1 indicates a net dehydration of the tree stem. See Figure S2 for calculation of SRR. Different types of lines (solid, dashed, dotted) represent SRR values of individual trees of the same species.

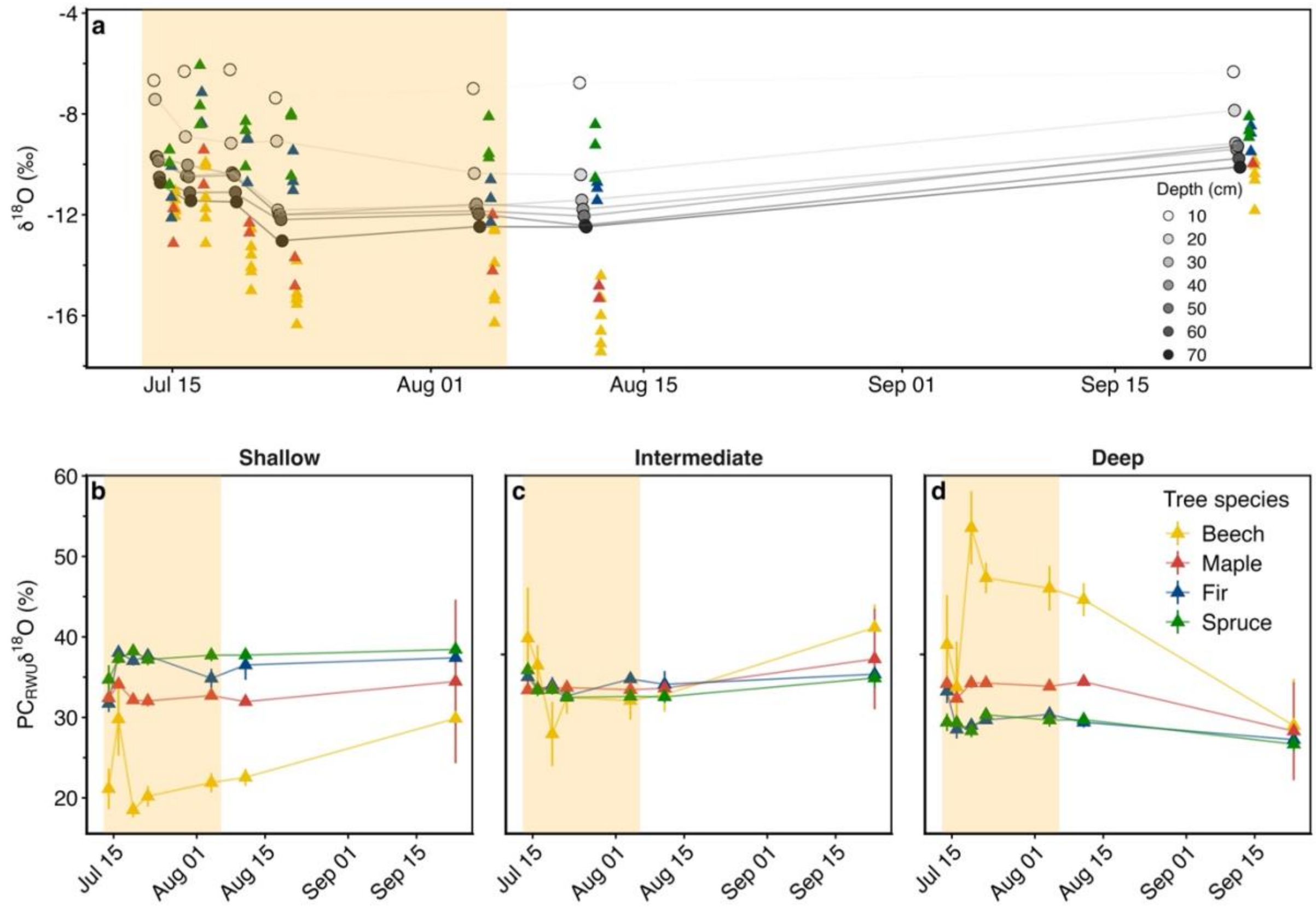

Figure S7. (a) Temporal variations of δ^{18}O of soil water from different depths (circles), and xylem water from four tree species (in triangles; beech, maple, fir, spruce). Soil water was extracted from bulk soil sampled at 0-10, 10-20, 20-30, 30-40, 40-50, 50-60, and 60-70 cm depth, with the lower range being used for the legend. Proportional contributions to root water uptake (PC_{RWU}) of (b) shallow (0-30 cm), (c) intermediate (30-70 cm) and (d) deep (> 70 cm) soil layers estimated based on δ^2H for four tree species using a Bayesian mixing model. Means and errors (± 95% C.I.) are given in b-d. The shaded yellow areas show the CSAD period at the Lägeren forest site (14th July to 4th August 2022).

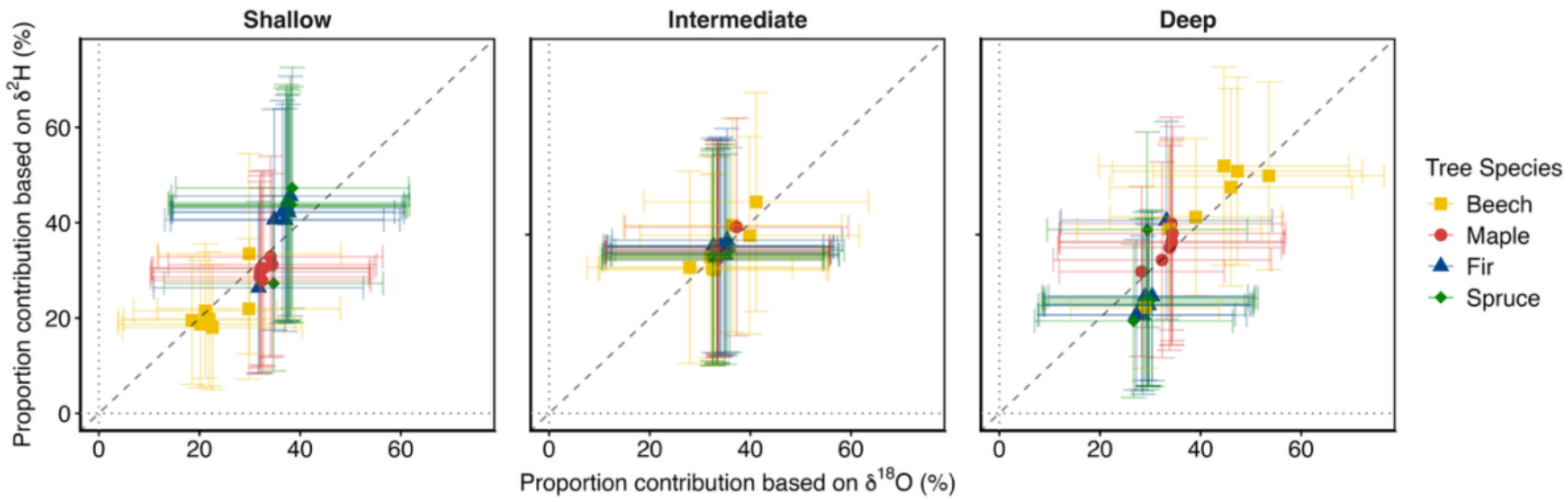

Figure S8. Comparison of the proportional contributions to root water uptake (PC_{RWU}) estimated based on $\delta^{18}O$ and $\delta^{2}H$ in shallow (0-30 cm), intermediate (30-70 cm) and deep (> 70 cm) soil layers for 14 trees of four tree species (beech, maple, fir, and spruce) over all the seven days of this study. The 1:1 line is shown by the dashed line. Error bars indicate standard deviations.

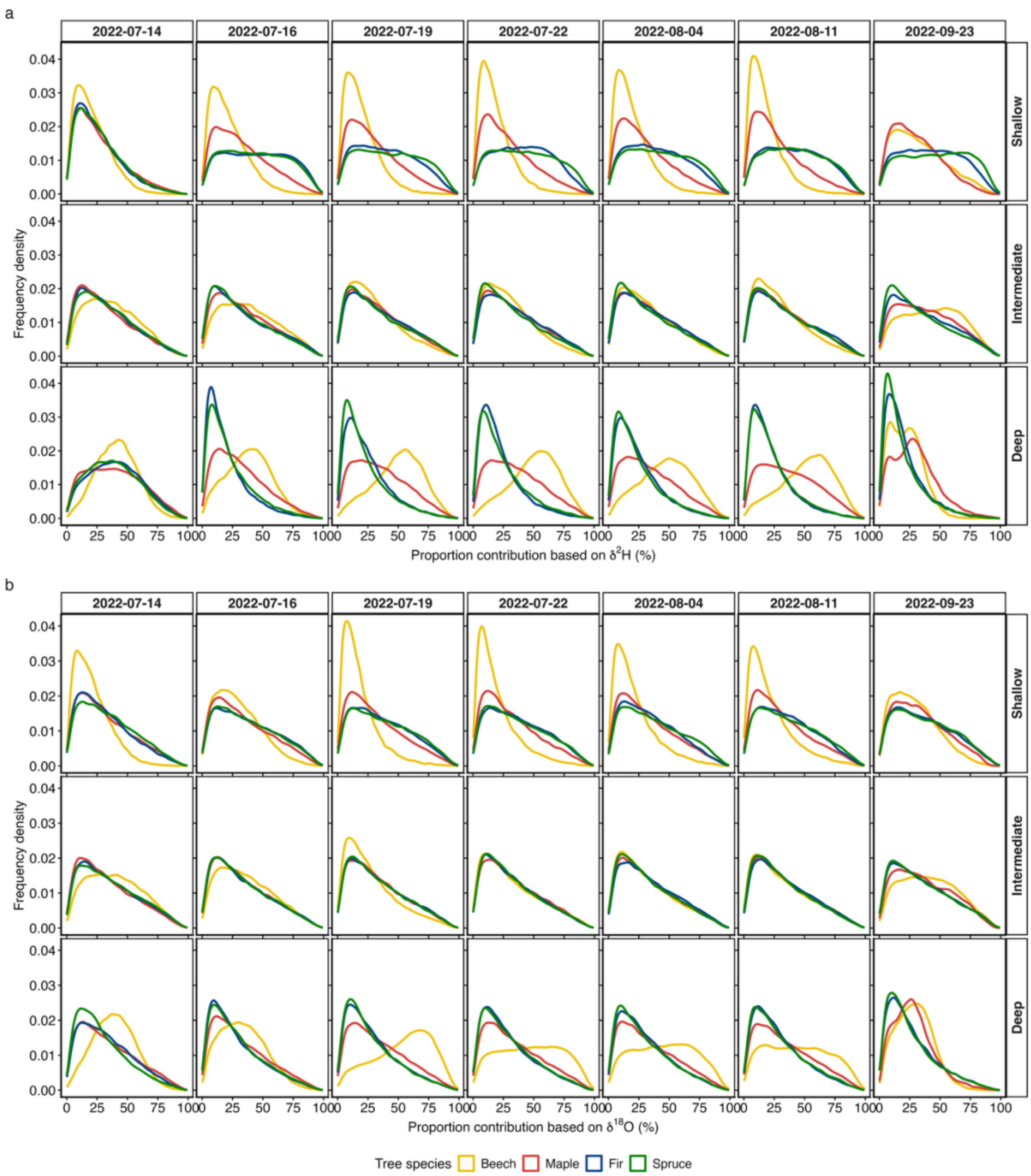

Figure S9. Frequency density plots of proportional contribution to root water uptake (PC_{RWU}) estimated based on δ^2H (a) and (b) δ^{18}O in shallow (0-30 cm), intermediate (30-70 cm) and deep (> 70 cm) soil layers for 14 trees of four tree species (beech, maple, fir, and spruce) over all the seven days of measurement.

Reference

Brinkmann, N., Eugster, W., Buchmann, N., Kahmen, A., 2019. Species-specific differences in water uptake depth of mature temperate trees vary with water availability in the soil. Plant Biol. 21, 71–81. https://doi.org/10.1111/plb.12907
